# Identification and bioinformatics analysis of a novel member of the lumbrokinase gene family in earthworms

**DOI:** 10.3389/fbinf.2026.1736746

**Published:** 2026-01-27

**Authors:** Xiong Rongchuan, Li Jianhong, Liu Tingchang, Su Chengyuan, Chen Hong, Chen Zhengpeng, Tang Chaozhi, Zhao Xinxia

**Affiliations:** School of Biological Science and Technology, Liupanshui Normal University, Liupanshui, Guizhou, China

**Keywords:** bioinformatics, cloning, earthworm, gene family, lumbrokinase

## Abstract

**Background:**

Lumbrokinase is a novel antithrombotic drug isolated and purified from earthworms and used in the treatment of stroke and cardiovascular disease. However, to date, there has been no systematic classification of lumbrokinase genes.

**Methods:**

In this study, a new member of the lumbrokinase gene family *LUKA*, which has not yet been reported, was cloned from *Eisenia andrei* by a homologous sequence method. The full-length cDNA sequence of *LUKA* was obtained through second-generation sequencing. The gene and protein were analyzed using various bioinformatics software tools.

**Results:**

The molecular formula of LUKA was C_1347_H_2068_N_360_O_419_S_18_, indicating it is a stable protein. The amino acid sequence of LUKA contained high proportion of valine (10.2%) and serine (9.2%) and possessed a peptide signal along with the corresponding cleavage site.

**Conclusion:**

Phylogenetic analysis showed that LUKA has multiple distinct amino acid sites compared with other lumbrokinases and represents a rare type of lumbrokinase. This study provides a new genetic material of rare lumbrokinase types.

## 1 Introduction

Earthworms belong to Opisthopora (Annelida: Oligochaeta) and comprise 3,500 terrestrial species worldwide, distributed across 12 families and 181 genera, of which 388 species (including subspecies) are found in China, belonging to 9 families and 31 genera (Xiao et al., 2023). As “ecosystem engineers” (Singh et al., 2016), the earthworms have various ecological functions, such as improving soil quality and increasing soil fertility (Wu, 2015; Chen et al., 2022), and they are widely used as biological indicators of organic pollution in soil (Fan et al., 2009; Yao et al., 2013). Meanwhile, earthworms, also known as “Di Long” in traditional Chinese medicine, are included in the earliest Chinese herbal medicine book, *Shen Nong Ben Cao Jing*, as a commonly used animal medicine, with functions such as clearing heat, calming the nervous system, promoting blood circulation, relieving asthma, and promoting urination. It has been used for thousands of years in China, and current research shows that lumbrokinase is one of the main active substances responsible for the anticoagulant activity of Di Long (Yang et al., 2017).

Lumbrokinase (LK), also known as earthworm fibrinolytic enzyme, is a group of proteases with fibrinolytic activity extracted from earthworms. LK can directly dissolve fibrinogen and fibrin, convert plasminogen to plasmin, and increase the activity of endogenous plasminogen activator (t-PA) in human tissue to dissolve fibrin clots and achieve anticoagulant effects (Li et al., 2012; Wang et al., 2021). It can also promote bone formation by promoting osteoblast activity and inhibiting osteoclast differentiation (Fu et al., 2014). In addition, LK can target the inactivation of the BPTF/VEGF and NF-κB/COX-2 in signaling pathways and be used in combination with bevacizumab and chemotherapeutic drugs to treat non-small cell lung cancer (Hua et al., 2024). Furthermore, LK also has antibacterial and wound healing effects (Wu, 2020).

There has not been a systematic study on the classification of *LK* gene family. In this study, we used *Eisenia andrei* (Bouché, 1972) as the experimental material and conducted second-generation sequencing. We compared the *LK* coding genes with those in GenBank and found a novel member of the *LK* gene family, the *LUKA* gene, which has not yet been reported. We analyzed the amino acid sequence LUKA and predicted species homology and physical and chemical properties, which paved the way for further developing rare LK types for clinical disease treatment and related theoretical research.

## 2 Methods

### 2.1 Experimental materials

Two earthworm specimens (sample no. LPSGS2022062604 and no. LPSGS2022062605) were collected from abandoned farmland on the edge of the desert in Minle County, Gansu Province (altitude: 1195 m), and were identified as *E. andrei* (Bouché, 1972).

### 2.2 Acquisition coding sequence of *LUKA*

Sample LPSGS2022062604 was taken from the anterior part of the body with clitellum, and sample LPSGS2022062605 was taken from the posterior part of the body without clitellum, both of which were frozen in liquid nitrogen, stored at −80 °C, and sent to Biomarker Biotechnology Co., Ltd. for transcriptome sequencing and assembly to obtain UniGene sequences. Then, the raw data (SRR35809842 and SRR35809843) were shared by uploading to GenBank. The UniGene sequences were then built into a local database, and the published *LK* homologous sequences (Table 1) were used as search conditions (queries) to obtain the *LUKA* coding sequences of *E. andrei* (Bouché, 1972) using BLASTn.

**TABLE 1:** Homologous sequence information of lumbrokinase used in this study.

Number	GenBank ID	Protein name	Number of amino acids	Molecular weight	Theoretical pI	Instability index	Aliphatic index	Grand average of hydropathicity	Number of MHC ligands	Number of high binders	Number of weak binders	Species
1	BAL43192	Fibrinolytic enzyme	281	30,597.37	5.10	37.49	78.72	−0.144	5	3	6	*Enchytraeus japonensis*
2	BAL43191	Fibrinolytic enzyme	281	30,609.44	5.79	37.64	76.98	−0.183	6	2	6	*Enchytraeus japonensis*
3	BAL43189	Fibrinolytic enzyme	281	30,683.47	5.61	36.86	77.33	−0.201	6	2	5	*Enchytraeus japonensis*
4	BAL43190	Fibrinolytic enzyme	281	30,658.37	5.19	39.52	77.33	−0.190	6	3	4	*Enchytraeus japonensis*
5	BAL43193	Fibrinolytic enzyme	281	30,496.16	5.28	37.31	77.05	−0.177	6	1	6	*Enchytraeus japonensis*
6	CAA11132	Chymotrypsin lumbricus	281	29,673.28	4.87	35.64	84.27	0.119	7	2	4	*Lumbricus rubellus*
7	T2204P LUKA	Lumbrokinase	283	30,586.48	4.82	30.00	78.13	−0.064	5	3	4	*Eisenia fetida*
8	T2205P LUKA	Lumbrokinase	281	30,342.13	4.82	30.14	77.30	−0.085	5	1	4	*Eisenia fetida*
9	AAR13224	Lumbrokinase-3 precursor	247	26,359.35	4.61	27.51	79.23	−0.083	6	0	4	*Eisenia fetida*
10	ABQ23217	Lumbrokinase partial	239	25,505.32	4.58	27.20	80.25	−0.060	6	0	4	*Eisenia fetida*
11	AAQ13828	Lumbrokinase-3T2 partial	239	25,500.30	4.57	24.99	80.67	−0.054	6	0	4	*Lumbricus rubellus*
12	AAA96502	Lumbrokinase-1T4 precursor	271	28,888.00	4.45	29.47	74.39	−0.149	7	0	4	*Lumbricus rubellus*
13	AAN28692	Lumbrokinase protein	271	28,915.02	4.45	28.60	74.39	−0.159	7	0	4	*Lumbricus rubellus*
14	AAL28118	Lumbrokinase lumbricus	271	28,842.02	4.50	29.16	75.46	−0.119	7	0	4	*bimastus*
15	BAB40768	Fibrinolytic enzyme	246	26,424.47	4.71	24.66	80.37	−0.084	6	0	4	*Lumbricus rubellus*
16	AAN78282	Lumbrokinase	246	26,486.46	4.64	23.44	78.78	−0.142	7	0	4	*Lumbricus bimastus*
17	AAA96503	Lumbrokinase-3 (1) precursor	270	28,976.29	4.64	27.05	76.85	−0.114	6	1	4	*Lumbricus rubellus*
18	AAL27616	Fibrinolytic enzyme partial	180	19,663.68	4.59	30.87	73.56	−0.250	5	0	4	*Eisenia fetida*
19	AXK90150	Fibrinolytic enzyme	245	26,417.54	4.82	29.81	77.51	−0.124	7	0	4	*Metaphire posthuma*
20	ATP16189	Lumbrokinase	245	26,210.31	4.79	27.29	77.96	−0.064	6	0	4	*Eisenia fetida*
21	ARD24433	Lumbrokinase precursor	245	26,209.37	4.97	28.26	77.96	−0.066	6	0	4	*Eisenia fetida*
22	1YM0_A	Fibrinolytic enzyme component B	238	25,315.18	4.50	27.43	78.99	0.013	7	0	4	*Eisenia fetida*
23	AIC77168	Lumbrokinase partial	238	25,456.39	4.58	28.42	80.63	0.000	7	0	4	*Perionyx excavatus*
24	ABB19359	Fibrinolytic protease P-III-1	238	25,456.39	4.58	28.42	80.63	0.000	7	0	4	*Eisenia fetida*
25	AHY19039	Fibrinolytic enzyme	245	26,209.32	4.61	28.78	79.92	−0.022	7	0	4	*Perionyx excavatus*
26	BAB40767	Fibrinolytic enzyme	245	26,155.18	4.61	29.01	77.14	−0.062	7	0	4	*Lumbricus rubellus*
27	QBA57435	Lumbrokinase	245	26,139.14	4.61	30.81	75.55	−0.084	7	0	4	*Bimastos parvus*
28	AAT74900	Lumbrokinase	246	26,297.38	4.68	26.57	78.01	−0.046	7	0	4	*Eisenia fetida*
29	AAT74899	Lumbrokinase	245	26,108.17	4.54	27.07	78.33	−0.029	7	0	4	*Eisenia fetida*
30	ABA43718	Lumbrokinase F238 precursor	245	26,169.21	4.61	29.96	77.55	−0.053	7	0	3	*Eisenia fetida*
31	ABW04903	Lumbrokinase partial	238	25,444.34	4.58	27.79	78.99	−0.022	7	0	4	*Eisenia fetida*

### 2.3 Phylogenetic analysis of LUKA

The obtained LUKA sequence was uploaded to GenBank for BLASTp, yielding 101 homologous protein sequences. To identify the *LK* family genes, the *LK-*containing proteins from 29 of 101 earthworm specimens were used to construct a phylogenetic tree, combined with two self-test sequences (*T2204P LUKA* and *T2205P LUKA*; NCBI accession numbers: PX515663 and PX515664) to form dataset A. No outgroup was specified during the phylogenetic analysis to explore the clustering relationships among sequences. After the construction of the phylogenetic trees, we identified a well-supported monophyletic group (ingroup) that contains the target sequences along with closely related homologous sequences. On the phylogenetic trees, we also identified a well-supported monophyletic group that is genetically distant from the ingroup, which we used as the outgroup to interpret the evolutionary relationships. The amino acid sequences were aligned using the Muscle tool in MEGA X software for phylogenetic tree construction. Neighbor-joining tree (NJ tree) analysis was performed with manual correction and the unpairwise deletion option based on P-distance. The ModelFinder in PhyloSuite software was used to select the optimal model for sequence evolution in the dataset, which was then used by IQ-TREE and MrBayes to construct the maximum likelihood tree (ML tree) and the Bayesian inference tree (BI tree), respectively. According to the Akaike information criterion (AIC), the optimal model for the dataset was GTR + I + G, with G = 0.885 and I = 0.605. If the Bayesian inference posterior probability was greater than or equal to 95%, it fully supports the branch. In NJ and ML trees, a bootstrap probability greater than or equal to 70% was considered to fully support the branch relationship, 50%–70% was considered moderate support, and values below 50% were regarded as unresolved.

### 2.4 Bioinformatics analysis of the *LUKA* gene and its encoded protein

The sequencing results of the obtained *LUKA* gene were subjected to transcript analysis using DNASTAR.Lasergene.v7.1 software. The amino acid sequence of LKs was analyzed using ProtParam (https://web.expasy.org/protparam/) in the ExPASy database; the signal peptide of LKs was predicted and analyzed using SignalP5.0 (https://services.healthtech.dtu.dk/services/SignalP-5.0/); the cleavage site of the LK signal peptide was predicted using ProP 1.0 (https://services.healthtech.dtu.dk/service.php?ProP-1.0); the subcellular localization of the LK signal peptide was predicted using PSORT (https://psort.hgc.jp/form2.html); the transmembrane domain of LKs was predicted using TMHMMl (https://services.healthtech.dtu.dk/services/TMHMM-2.0/); the glycosylation distribution of LKs was predicted using the software program DictyOGlyc 1.1 (https://services.healthtech.dtu.dk/service.php?DictyOGlyc-1.1); the phosphorylation distribution of LKs was predicted using NetPhos 3.1a (https://services.healthtech.dtu.dk/service.php?NetPhos-3.1); the affinity/hydrophobicity of LKs was predicted using ProtScale (https://web.expasy.org/protscale/); and the major histocompatibility complexes (MHCs) of LKs were predicted using NetCTL 1.2 (https://services.healthtech.dtu.dk/service.php?NetCTL-1.2) and NetMHC-4.0 (https://services.healthtech.dtu.dk/service.php?NetMHC-4.0). The enzymatic activity of LKs was identified using the PROSITE database (https://prosite.expasy.org/scanprosite/). Homologous modeling of the LK protein was performed using SWISS-MODEL (https://swissmodel.expasy.org/interactive).

## 3 Results

### 3.1 Acquisition of the full-length sequence of *LUKA*

**FIGURE 1:**
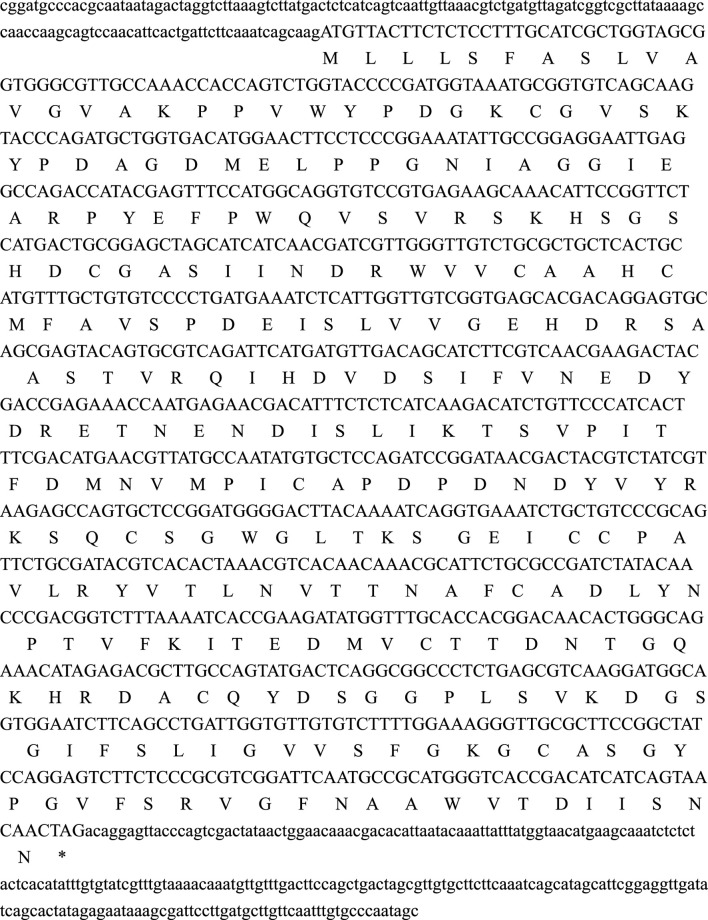
Lumbrokinase gene *LUKA* encodes a protein with 283 amino acids.

The second-generation sequencing results of the collected earthworm tissue samples were analyzed using DNASTAR.Lasergene.v7.1. The results showed that the full-length cDNA sequence of the *LUKA* gene (*T2204P LUKA*, an entirely amplified DNA fragment) is 1,233 bp, the open reading frame of it is 852 bp, and it encodes 283 amino acids (Figure 1).

### 3.2 Phylogenetic tree construction and homology analysis of LK

**FIGURE 2:**
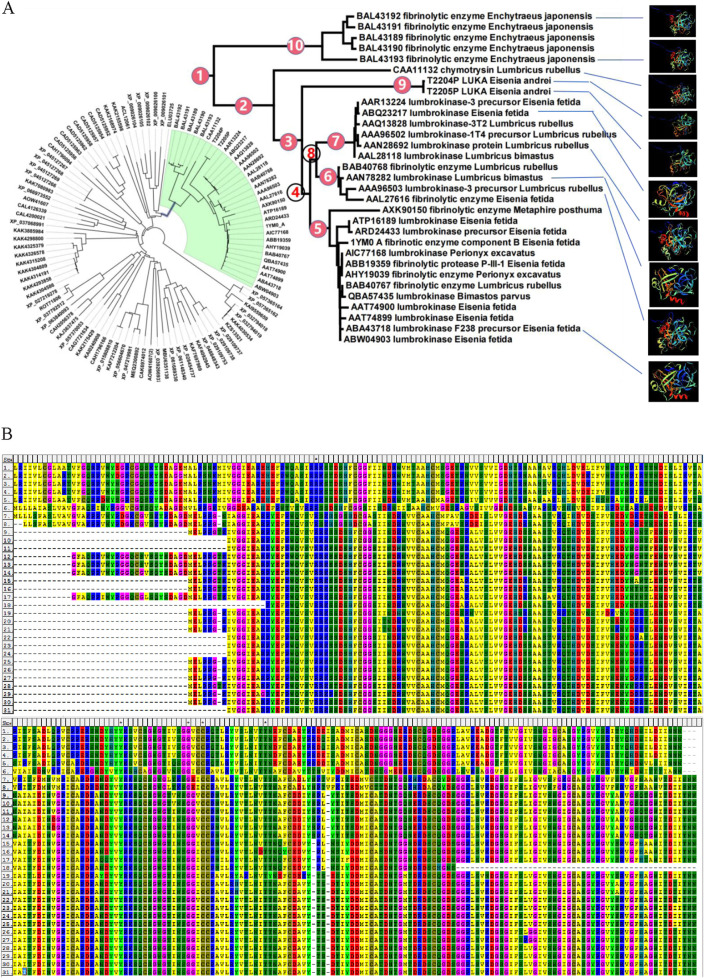
LUKA clusters into a new independent branch in the phylogenetic tree. **(A)** In the NJ tree, constructed according to homologous protein sequences of LKs, the number next to the branch is the node number; the solid circle indicates that the branch is highly supported in the neighbor-joining, Bayesian, and ML trees; and the hollow circle indicates that the support rate of the branch is low in the three phylogenetic trees. **(B)** Alignment of homologous amino acid sequences of LKs.

The amino acid sequences of LK cluster into a branch (clade three) with a high support rate, which further divides into four branches (clades five, six, seven, and nine), each with high support values. The four branches are independent and complementary clustering. Clades five, six, and seven all have homologous LK sequences from *Eisenia fetida* and *Lumbricus rubellus*, and clades six and seven have homologous LK sequences from *Lumbricus bimastus*. That is, homologous sequences of LK from the same species are distributed in independent branches (Figure 2A), suggesting that the four branches may be different members of the LK family. The two sequences obtained in this study cluster into a new independent branch (Figure 2A), indicating that *LUKA* is a new member gene in the *LK* family.

LUKA (T2204P LUKA and T2205P LUKA) has two amino acid variant sites compared to other LKs. If the first amino acid of LUKA (T2204P LUKA) is defined as No. 1, the amino acid at No. 23 changes from G to D and No. 232 changes from G to Y (Figure 2B).

### 3.3 Analysis of the physical and chemical properties of LUKA

The predicted molecular formula of LUKA (T2204P LUKA), analyzed using the ProtParam software program in the ExPASy database, is C_1347_H_2068_N_360_O_419_S_18_, consisting of 283 amino acids, with a predicted molecular weight of 30,586.48 and a theoretical isoelectric point (PI) of 4.82. The amino acid sequence of LUKA (T2204P LUKA) has 33 negatively charged residues (Asp + Glu) and 21 positively charged residues (Arg + Lys) and a high content of valine (10.2%) and serine (9.2%). LUKA (T2204P LUKA) is predicted as a stable protein with an instability index of 30.00. LUKA (T2204P LUKA) has an aliphatic index of 78.13 and a grand average of hydropathicity (GRAVY) of −0.064, and its estimated half-life *in vitro* of mammalian reticulocytes is 30 h.

**FIGURE 3:**
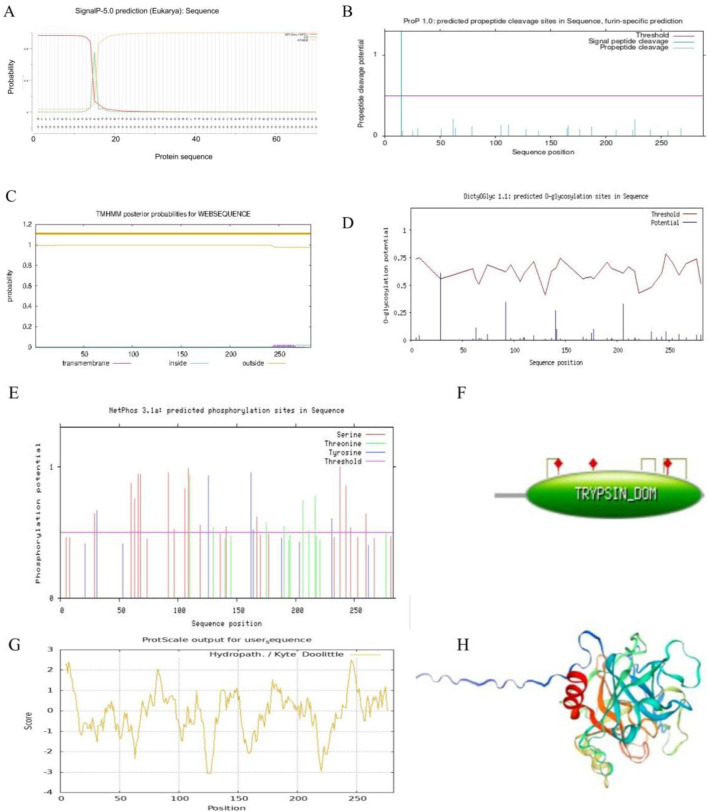
Bioinformatics analysis of lumbrokinase LUKA. **(A)** Prediction of signal peptides of the selected LUKA sequences; **(B)** prediction of cleavage sites of the selected LUKA; **(C)** prediction of the transmembrane domain of LUKA; **(D)** prediction of the glycosylation distribution of LUKA; **(E)** prediction of the phosphorylation distribution of LUKA; **(F)** enzyme activity identification of LUKA; **(G)** prediction of the affinity/hydrophobicity of LUKA; **(H)** homology modeling structure diagrams of the 31 selected lumbrokinase sequences.

The prediction results of the signal peptide, analyzed using Signal P-5.0, showed that the proportion of SP(Sec/SPI) is 0.9632 and the proportion of other sequences is 0.0358. The signal peptide is located in the first 16 amino acids at the N-terminal of LUKA (T2204P LUKA) (Figure 3A). The cleavage site of LUKA (T2204P LUKA), predicted using ProP 1.0, is located around the 20th amino acid in the N-terminal region (Figure 3B), which is consistent with the signal peptide predicted using Signal P-5.0.

We focused on the N-terminal sequences of LUKAs and further predicted the signal peptides of the 31 selected LK sequences. The results showed that the LK sequences located in clade three all had signal peptides with higher probability (more than 0.6), and the LUKA (T2204P LUKA and T2205P LUKA) also had similar signal peptide sequences (Supplementary Figure S1). None of the other sequences predicted a signal peptide sequence. The prediction results of signal peptide cleavage sites showed that the LKs with signal peptides predicted the signal peptide cleavage sites at the corresponding regions. In contrast, the other sequences were not expected, indicating that LUKA (T2204P LUKA and T2205P LUKA) may have a clearly subcellular localization with the LK sequences in clade three (Supplementary Figure S2).

Subcellular localization prediction was performed for the 31 selected LK sequences. The results showed that the LK sequences with the signal peptides in clades three and nine were localized extracellularly and in organelles such as mitochondria and Golgi, while the LK sequences without the signal peptides were mainly distributed in the cytoplasm (Supplementary Table S1).

Proteins with signal peptides are usually localized to plasma membranes (Li et al., 2000), 31 LK sequences are selected for transmembrane domain prediction, and the results show that except for CAA11132 in clade three, which has two highly probable transmembrane domains at the N-terminal and the C-terminal, the other LK sequences have only one or no predicted transmembrane domain at the C-terminal. The sequence QBA57435 in clade five does not predict a transmembrane domain, while other sequences in the same branch have a transmembrane domain at the C-terminal. AAL27616 (the shortest peptide chain) and LUKA (neither T2204P LUKA nor T2205P LUKA) were not predicted to have a transmembrane domain (Figure 3C; Supplementary Figure S3).

LK is a protein formed by the condensation of multiple amino acids, which can only be used by intravenous injection, and its water solubility should be considered when choosing LK to dissolve thrombus (Lai et al., 2025). Through the hydrophilicity prediction of 31 selected LK sequences, the results showed that the LK sequence CAA11132 in clade three was significantly different from the other LK sequences in the same branch, and its GRAVY was 0.119. By examining the total sequence amino acid distribution, the high GRAVY may be due to the high distribution of hydrophilic amino acids at the N-terminal (Table 1; Supplementary Figure S4). In this study, the distribution of hydrophilic and hydrophobic amino acids in the sequence of LUKA (T2204P LUKA and T2205P LUKA) was roughly equal, and the GRAVY was relatively negative (Table 1; Figure 3G).

Glycosylation and phosphorylation are crucial in protein function (Kratka et al., 2025). One glycosylation site is predicted around No. 30 amino acid at the N-terminal of LUKA (T2204P LUKA) using DictyOGlyc 1.1 (Figure 3D). Meanwhile, glycosylation sites were predicted for the other 29 selected LK sequences, and the results showed that, except for the four LK sequences in clade six, ATP16189, ARD24433, and 1YM0_A, the glycosylation sites were predicted in the remaining sequences. Except for the sequences BAL43192, BAL43191, BAL43189, and BAL43190 in clade three, which have two glycosylation sites, most of the remaining sequences predict only one glycosylation site (Supplementary Figure S5). Three types of amino acids are predicted to be phosphorylated using NetPhos 3.1a: Serine (Ser) and tyrosine (Tyr) are distributed throughout the entire amino acid sequence of LUKA (T2204P LUKA and T2205P LUKA). In contrast, threonine (Thr) is mainly distributed at the C-terminal of LUKA (T2204P LUKA and T2205P LUKA) (Figure 3E). In addition, phosphorylation sites were also predicted for the other 29 selected LK sequences, and the results (Supplementary Figure S6) showed that, compared with the LUKA (2204P LUKA and T2205P LUKA) obtained in this study, each LK sequence had multiple phosphorylation sites, and serine was the primary phosphorylation site in all of them.

The number of MHCs varies in different species in the same family (Grace et al., 2024). Five MHC ligands are predicted in LUKA (T2204P LUKA) using NetCTL 1.2, of which, two MHCs arrive at an extremely significant level and three at a significant level (Supplementary Table S2). Furthermore, NetMHC-4.0 was used to predict the binding peptides of LUKA (T2204P LUKA) for MHC-I molecules and protein sequences, and three strongly binding peptides and four weakly binding peptides were obtained. Meanwhile, a tryptic activity across amino acids 44–281 in LUKA (T2204P LUKA) identified by the PROSITE database, and it has three activation sites, namely, 86 histidine (His), 134 aspartate (Asp), and 233 serine (Ser). Furthermore, three disulfide bridges are predicted in this region, which are between amino acids 71–87, 199–216, and 229–258, respectively (Figure 3F). Further enzymatic activity assays of the other selected 29 LK sequences showed that, including the shortest peptide chain AAL27616 (with only 180 aa), all were predicted to have trypsin domains and similar disulfide bond modifications (Supplementary Figure S7).

Finally, LUKA (T2204P LUKA and T2205P LUKA) was modeled by homology using SWISS-MODEL software. The results showed that the first 15 amino acids at the N-terminal form a finger-like structure, far away from the core domain of LUKA (T2204P LUKA), which is consistent with the functional domain prediction of LUKA (T2204P LUKA) (Figure 3F) and the signal peptide and corresponding cleavage site prediction of LUKA (T2204P LUKA) (Figures 3A,B). Homology modeling was conducted on the other 29 selected LK sequences, and the results indicated that the LKs in clade three all had extended peptide chain at the N-terminal, except for AAA96503 in clade six and AAA96502, AAN28692, and AAL28118 in clade seven, which had relatively short peptide chain extensions. The LUKA (T2204P LUKA and T2205P LUKA) cloned in this study had a peptide chain extension similar to those in clade 3 (Supplementary Figure S8).

## 4 Discussion

LK is a type of multifunctional biomedical protein. *LUKA* is an *LK* gene that we report for the first time. In this study, the full-length cDNA sequence of the *LUKA* gene was obtained through transcriptome sequencing. Phylogenetic tree analysis showed that the amino acid sequence of LUKA differs substantially from those of existing LKs, indicating that it represents a relatively rare type. This research enriches candidate materials for obtaining more effective LKs. LUKA was predicted to possess a signal peptide of approximately 16 amino acids at the N-terminal, with a corresponding cleavage site, which is also consistent with predictions of the functional domains and three-dimensional structure of LUKA, indicating that LUKA has precise subcellular localization after synthesis through the signal peptide. No transmembrane domains were predicted for LUKA (T2204P), suggesting that it is not a membrane protein. This is consistent with the predicted primary subcellular localization of LUKA (T2204P) as extracellular, suggesting that LUKA synthesized by earthworm cells is secreted into the intestine to decompose the ingested residue. The proportion of LUKA predicted to be transported extracellularly after synthesis was relatively high, at 55.6%, suggesting that it may be a lumbrokinase with a strong dissolving activity (Supplementary Figure S1).

It has been previously reported that phosphorylation regulates the activity of proteases (Lin et al., 2015; Deng et al., 2021). Mutagenesis analysis found that T_44_ and T_150_, two key amino acid residues related to 14-3-3 binding, are substrates of AKT kinase (Shi, 2019). Meanwhile, glycosylation modification of drug proteins plays a crucial role in the function of pharmaceutical proteins (Zhang et al., 2020). The phosphorylation sites are mainly distributed at the C-terminal of LUKA, suggesting that this region may play an important role in regulating LUKA activity and may be a primary target for studying the action mechanism of this enzyme and improving the efficiency of LKs. Only one potential glycosylation site was identified at the N-terminal of LUKA, which represents a key site for the humanized modification of LUKA.

The MHC is closely associated with many diseases. For example, a TM-score for predicting immunotherapy efficacy and overall survival (OS) in patients with gastric cancer has been established by combining tumor mutation burden (TMB) and MHC, and it has been found that TMB, MHC-I, and MHC-II are protective factors in patients with gastric cancer (Xiang, 2023). MHC-II is a core component of the antigen presentation pathway, and its function is regulated by estrogen, which can promote T-cell-mediated production of various inflammatory factors by participating in adaptive immune responses mediated by T and B lymphocytes, ultimately promoting bone formation and osteoclast-mediated bone resorption (Zhang et al., 2024). As an essential component of the chicken immune system, MHC is mainly responsible for presenting antigen epitopes to specific T lymphocytes and inducing immune responses (Jia et al., 2024). However, MHC is highly complex, which increases the difficulty of developing disease-associated mutants (Sun et al., 2022). In this study, five MHC ligands are predicted in LUKA, yielding three strongly binding peptides (including one in T2205P LUKA) and four weakly binding peptides. The LUKA reported for the first time in the study enriches candidate materials for research on the use of LKs to treat diseases through the MHC pathway.

## 5 Conclusion

In this study, the full-length cDNA sequence of gene *LUKA* was amplified first, and its encoded protein was analyzed using various bioinformatics software programs. The amino acid sequence of LUKA differs from those of existing LKs, which is a relatively rare type. It provides the research material for the follow-up related research.

## Data Availability

The raw data supporting the conclusions of this article will be made available by the authors, without undue reservation.

## References

[B1] Chen J. Chen J. J. Yang Q. N. Cevin T. Li X. D. Zhang X. L. (2022). Effects of earthworm ecotype and density on comprehensive quality of watered land soil. J. South. Agric. 53 (07), 1899–1907.

[B2] Deng R. Zhu X. F. Yu Y. Li Z. L. Mai J. Peng X. D. (2021). Molecular mechanisms and clinical implications of c-Met-mediated Fis1 phosphorylation induces mitochondrial fission to facilitate tumor metastasis in liver cancer. Sun Yat-Sen University Cancer Center.

[B3] Fan Y. W. Zhou Q. X. Wang Y. Y. Zhu S. (2009). Toxic effects of BTEX in water on Daphnia magna and Limnodrilus hoffmeisteri and safety assessment of the aquatic environment. Acta Sci. Circumstantiae 29 (07), 1485–1490.

[B4] Fu Y. T. Chen K. Y. Chen Y. S. Yao C. H. (2014). Earthworm (Pheretima aspergillum) extract stimulates osteoblast activity and inhibits osteoclast differentiation. BMC Complementary Altern. Med. 14, 440. 10.1186/1472-6882-14-440 25387689 PMC4233063

[B5] Grace D. Kate R. Andrew O. Marina T. S. Beata U. (2024). Organisation and evolution of the major histocompatibility complex class I genes in cetaceans. IScience 27, 109590. 10.1016/j.isci.2024.109590 38632986 PMC11022044

[B6] Hua C. Y. Guo Z. Y. Dai M. Zhou J. Ge H. X. Xue G. Q. (2024). Lumbrokinase extracted from earthworms synergizes with bevacizumab and chemotherapeutics in treating non-small cell lung cancer by targeted inactivation of BPTF/VEGF and NF-κB/COX-2 signaling. Biomolecules 14, 741. 10.3390/biom14070741 39062456 PMC11274885

[B7] Jia Y. S. Liao M. Dai M. M. (2024). Research progress on the relationship between the molecular structure of chicken MHC and disease resistance. China Animal Husb. and Veterinary Med. 51 (01), 242–254.

[B8] Kratka K. Sistik P. Olivkova I. Kusnierova P. Svagera Z. Stejskal D. (2025). Mass spectrometry-based proteomics in clinical diagnosis of amyloidosis and multiple myeloma: a review (2012-2024). J. Mass Spectrom. 60 (3), e5116. 10.1002/jms.5116 39967472 PMC11836596

[B9] Lai X. Qiao J. Liu J. Zhou X. Zhang C. Peng Q. (2025). Albumin as a functional carrier enhances solubilization, photodynamic and photothermal antibacterial therapy of curcumin. Int. J. Biol. Macromol. 303, 140759. 10.1016/j.ijbiomac.2025.140759 39919397

[B10] Li Y. Luo L. Thomas D. Y. Kang C. Y. (2000). The HIV-1 Env protein signal sequence retards its cleavage and down-regulates the glycoprotein folding. Virology 272 (2), 417–428. 10.1006/viro.2000.0357 10873786

[B11] Li G. Q. Wang K. Y. Li D. H. Wang N. Liu D. (2012). Cloning, expression and characterization of a gene from earthworm *Eisenia fetida* encoding a blood-clot dissolving protein. PLoS ONE 7 (12), e53110. 10.1371/journal.pone.0053110 23300872 PMC3531398

[B12] Lin C. Ren L. L. Jiang Y. He F. C. (2015). Mouse liver phosphoproteome methodology optimization and kinase analysis. Mil. Med. Sci. 39 (06), 407–412.

[B13] Shi Z. (2019). Study on the role and mechanism of cell cycle regulation by phosphorylation of Cables1 protein by AKT kinase in tumor therapy. Jinan University.

[B14] Singh S. Singh J. Vig A. P. (2016). Earthworm as ecological engineers to change the physico-chemical properties of soil: soil vs vermicast. Ecol. Eng. 90, 1–5. 10.1016/j.ecoleng.2016.01.072

[B15] Sun Y. Y. Yuan F. Wang L. Dai D. F. Zhan Z. J. Liang F. (2022). Recombination and mutation shape variations in the major histocompatibility complex. J. Genet. Genomics 49 (12), 1151–1161. 10.1016/j.jgg.2022.03.006 35358716

[B16] Wang Y. Xu X. W. Zhao X. Yin Z. N. (2021). Functionalized polymeric hybrid micelles as an efficient nanotheranostic agent for thrombus imaging and thrombolysis. Acta Biomater. 122 (122), 278–290. 10.1016/j.actbio.2020.10.015 33359293

[B17] Wu D. (2015). Effects of inoculating earthworm to vegetable field on Soil,Plant and environment following organic amendments. Nanjing Agricultural University.

[B18] Wu Y. L. (2020). Isolation, identification and mechanism study of antithrombotic component DPf3 from Trichotricha wilhelmii based on multi-omics model. Beijing University of Chinese Medicine.

[B19] Xiang K. H. (2023). A score based on tumor mutation load and major histocompatibility complex was constructed to predict immunotherapy efficacy and overall survival of gastric cancer. China Medical University.

[B20] Xiao N. W. Xu Q. Gao X. Q. Guo N. N. (2023). Earthworms in China. Beijing: Science Press.

[B21] Yang X. Liu X. Wan M. Zhang T. (2017). Research status on Pheretima anticoagulant active components. J. Jianghan Univ. Sci. Ed. 45 (01), 83–88.

[B22] Yao H. W. Zhu J. Shen G. X. (2013). Acute toxic effects of the perfluorooctane sulfonate pollutant on the earthworm Eisenia foetida. J. Saf. Environ. 13 (01), 1–4.

[B23] Zhang W. J. Wang S. Kang C. Z. Lv C. G. Zhou L. Huang L. Q. (2020). Pharmacodynamic material basis of traditional Chinese medicine based on biomacromolecules: a review. Plant Methods 16, 26. 10.1186/s13007-020-00571-y 32140174 PMC7049221

[B24] Zhang Z. Chen H. Wang X. P. Zhu L. L. (2024). The relationship between major histocompatibility complex class II molecules and postmenopausal osteoporosis. Chin. J. Osteoporos. 30 (02), 270–274.

