# Supplementary material for: Identification and bioinformatics analysis of a novel member of the lumbrokinase gene family in earthworms
Source: Front Bioinform. 2026 Jan 27;6:1736746. doi: 10.3389/fbinf.2026.1736746 (PMC12886433; doi:10.3389/fbinf.2026.1736746)
Supplement: Supplementary file 1 [file Supplementaryfile1.doc]

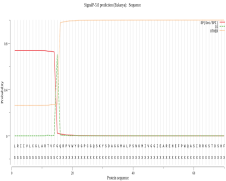

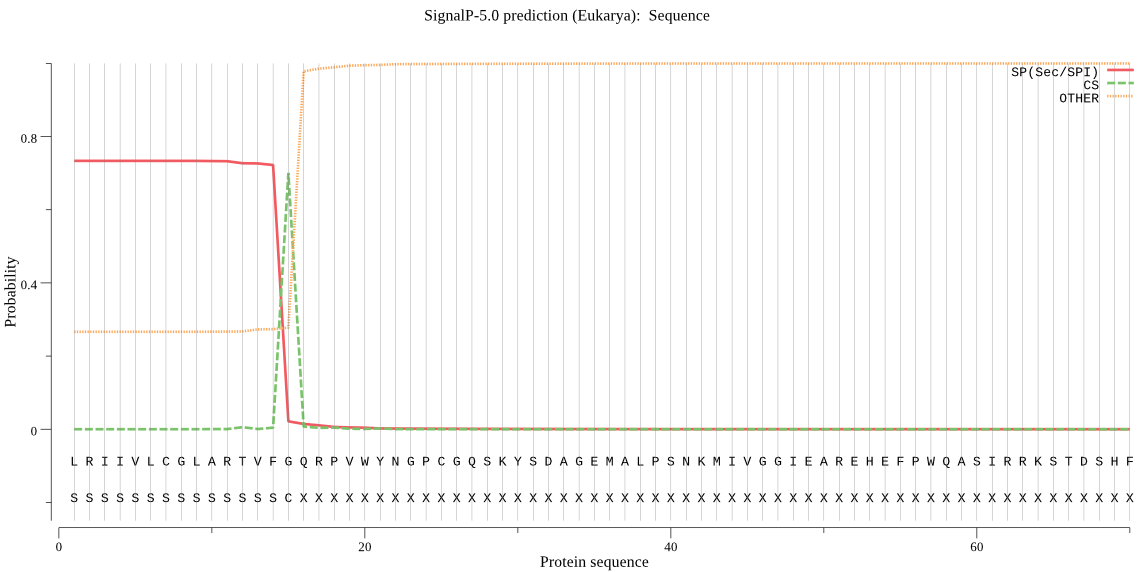

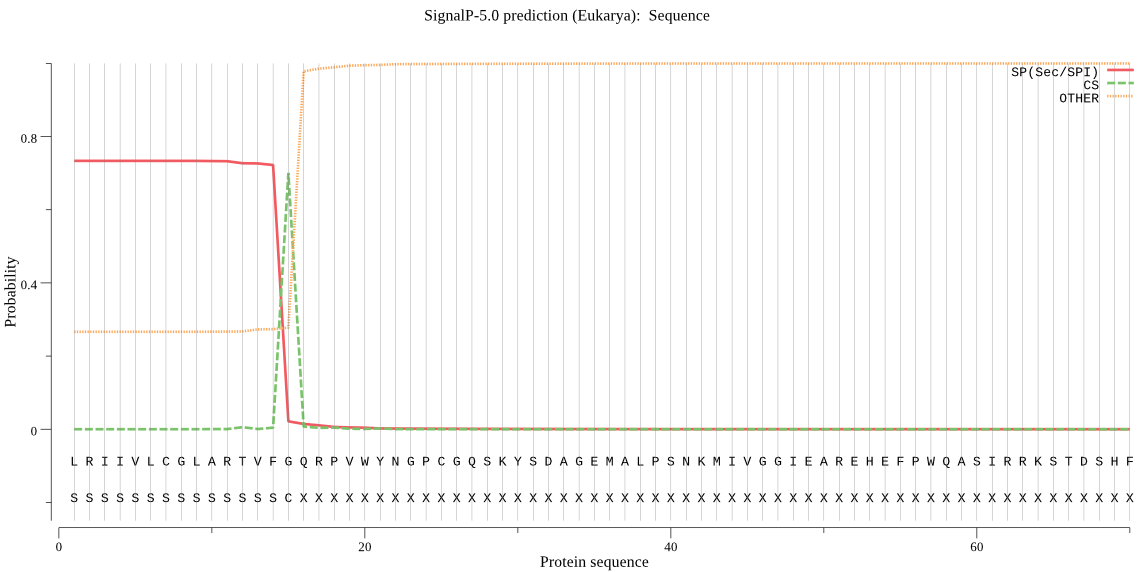

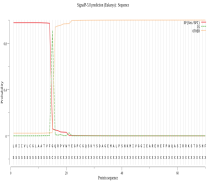


(1)

(2)

(3)

(4)


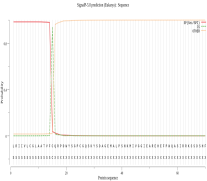

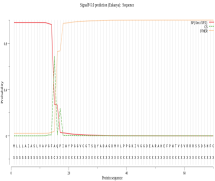

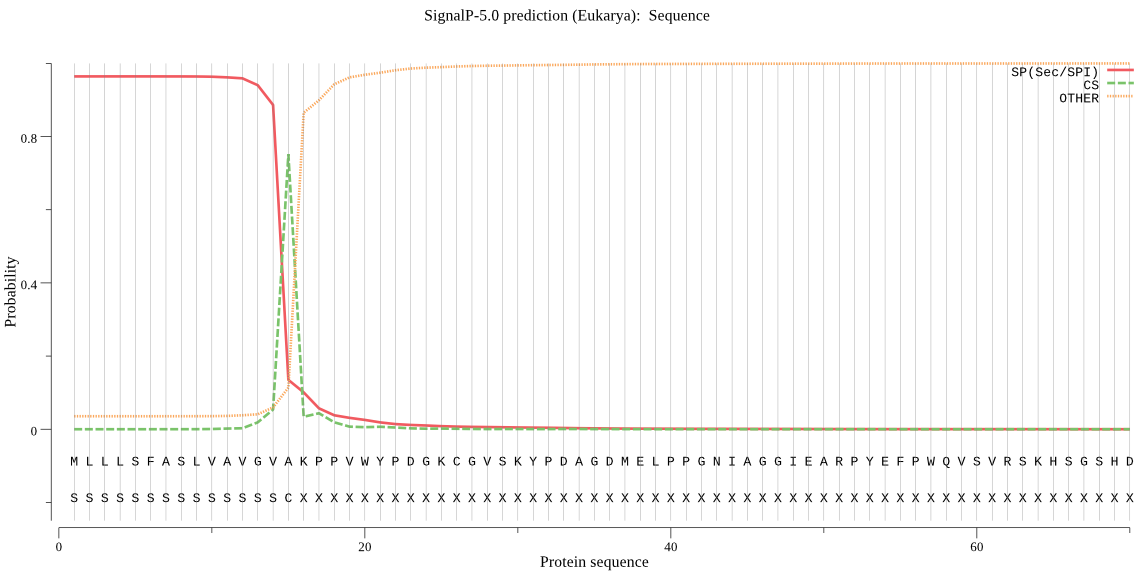

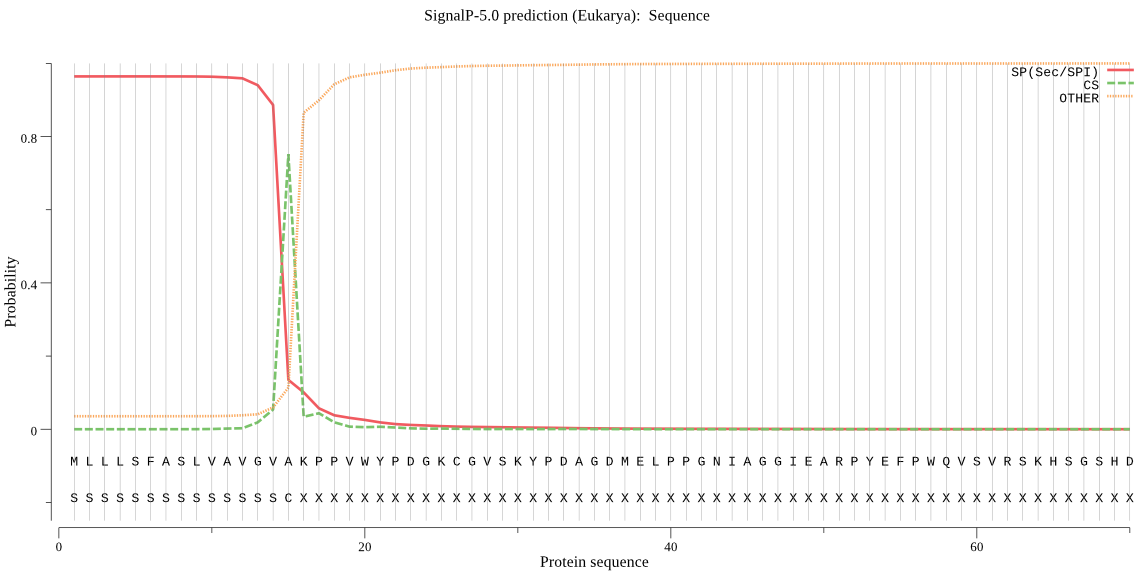


(5)

(6)

(7)

(8)


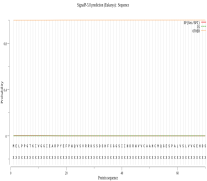

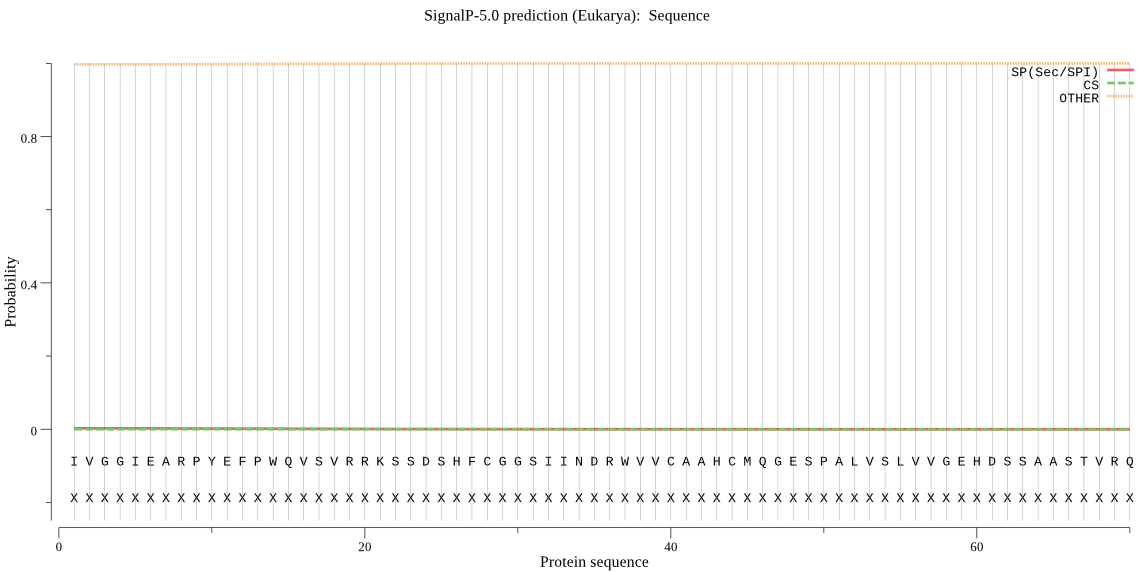

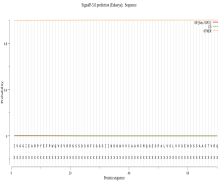

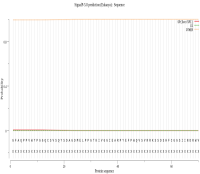


(9)

(10)

(11)

(12)


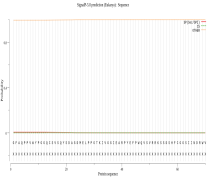

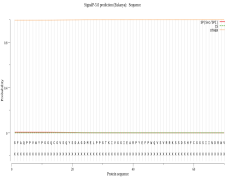

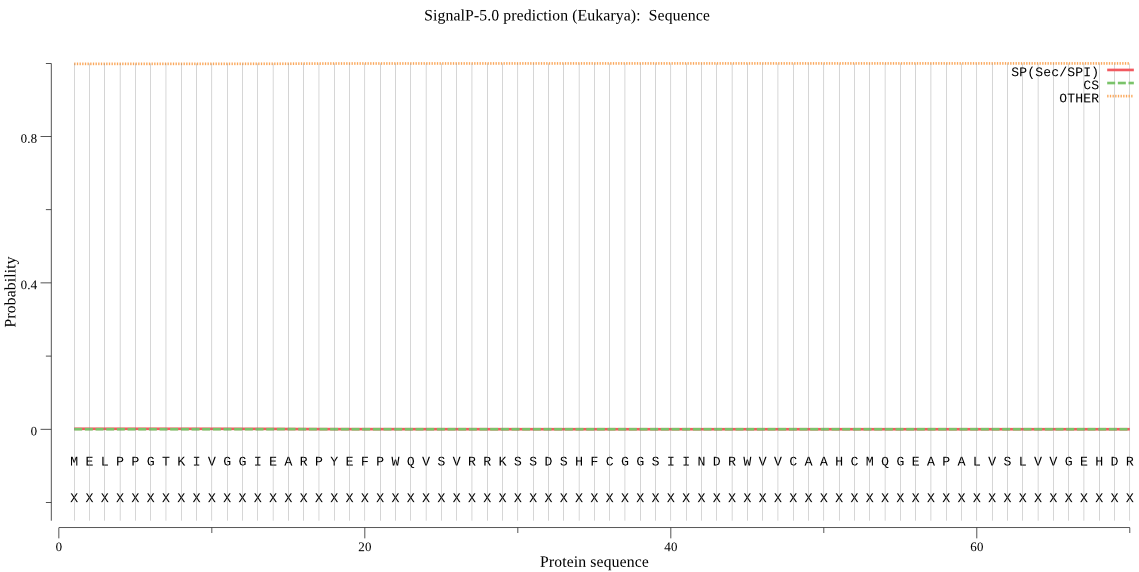

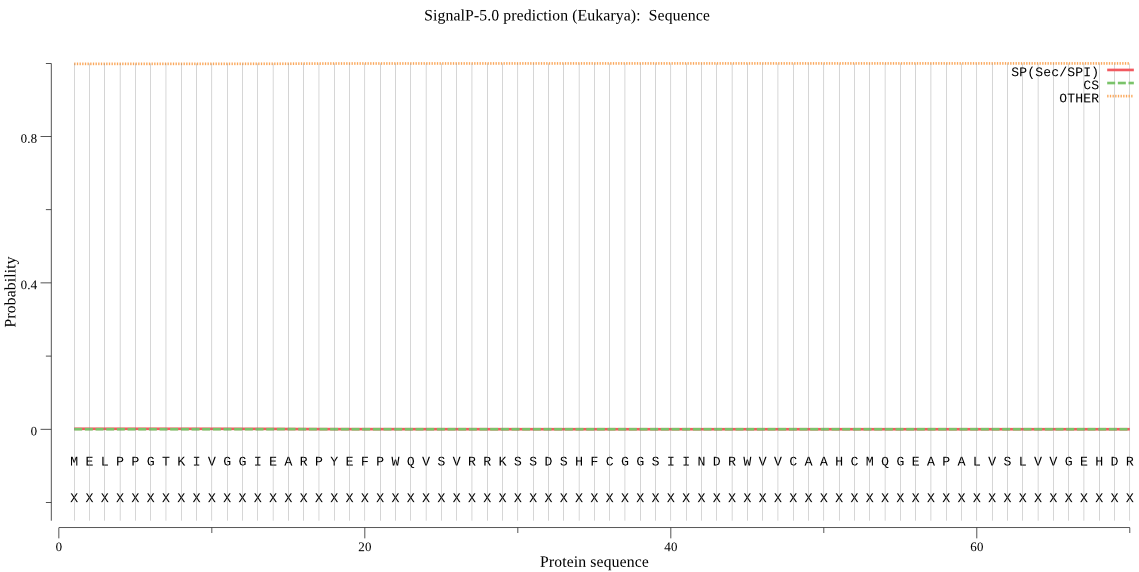


(13)

(14)

(15)

(16)


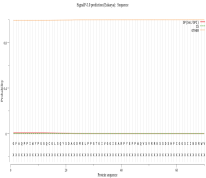

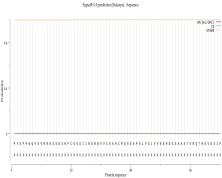

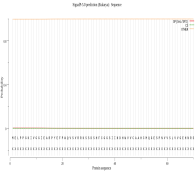

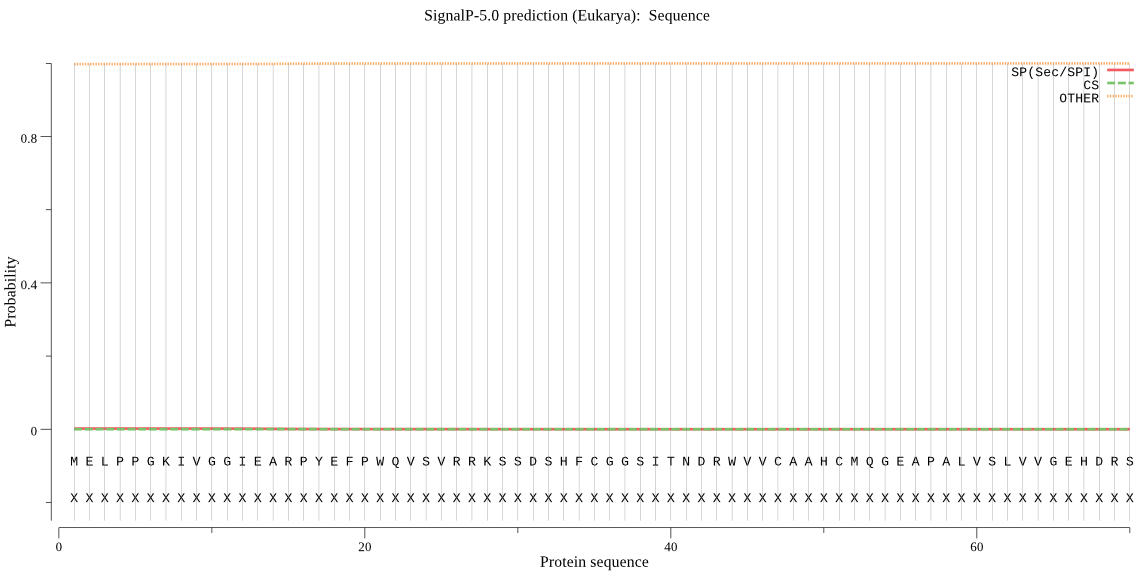


(17)

(18)

(19)

(20)


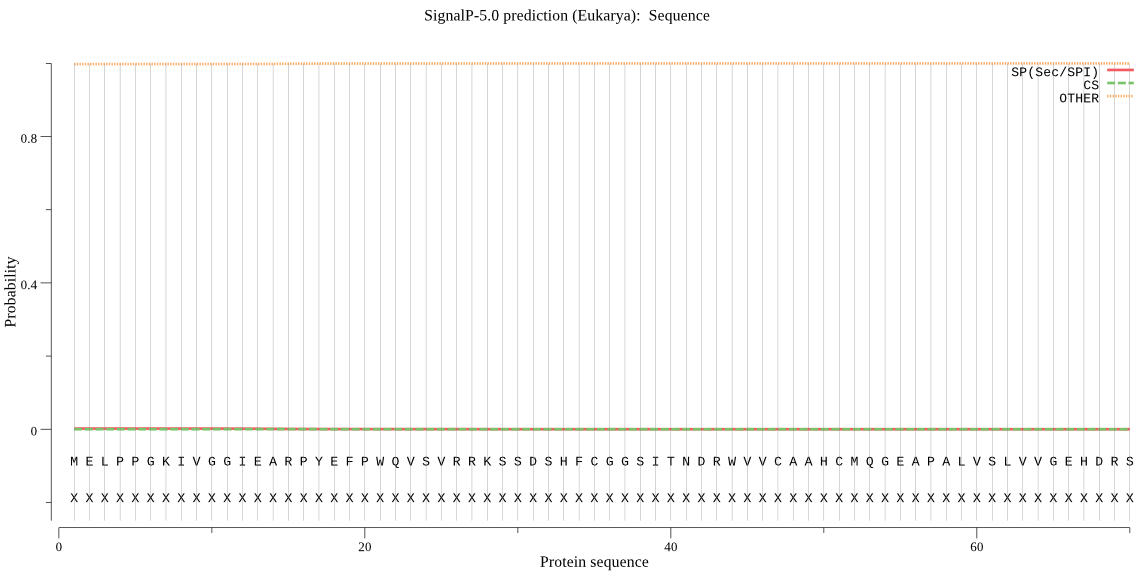

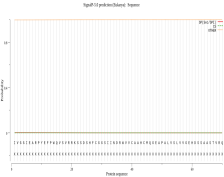

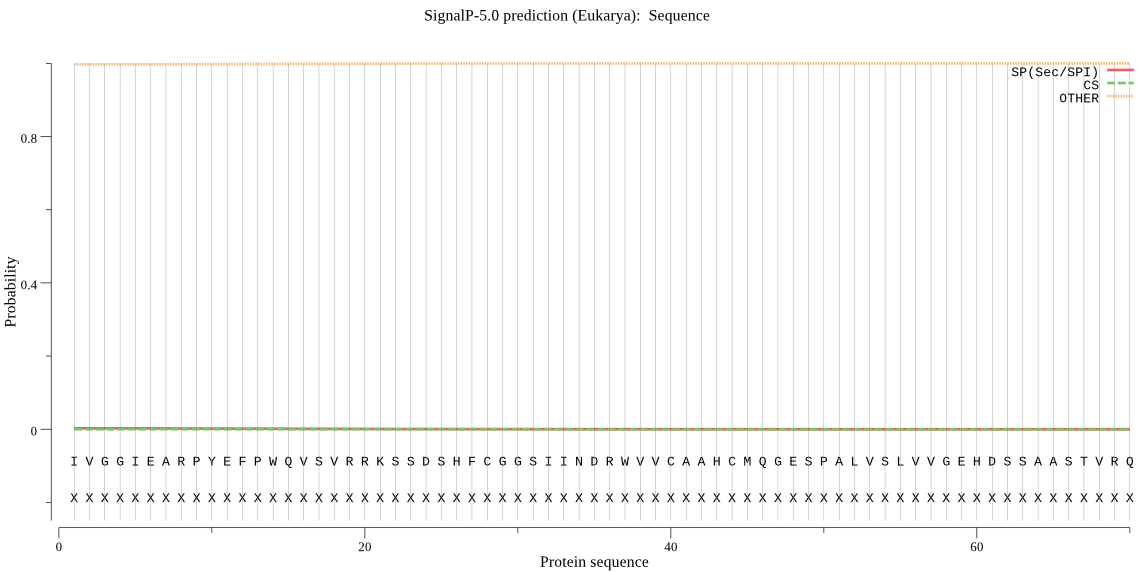

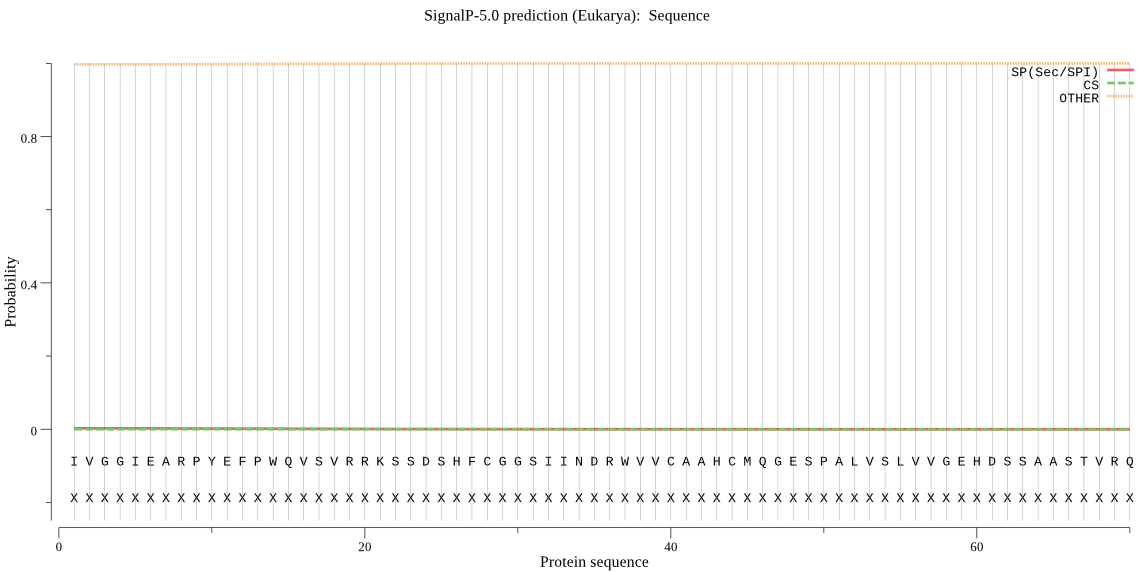


(21)

(22)

(23)

(24)


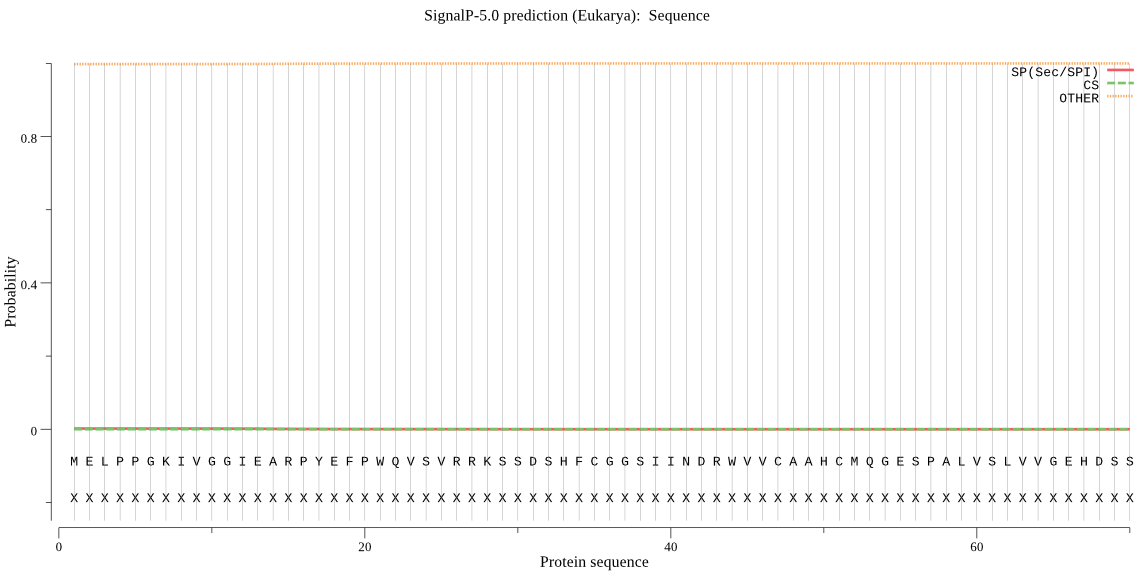

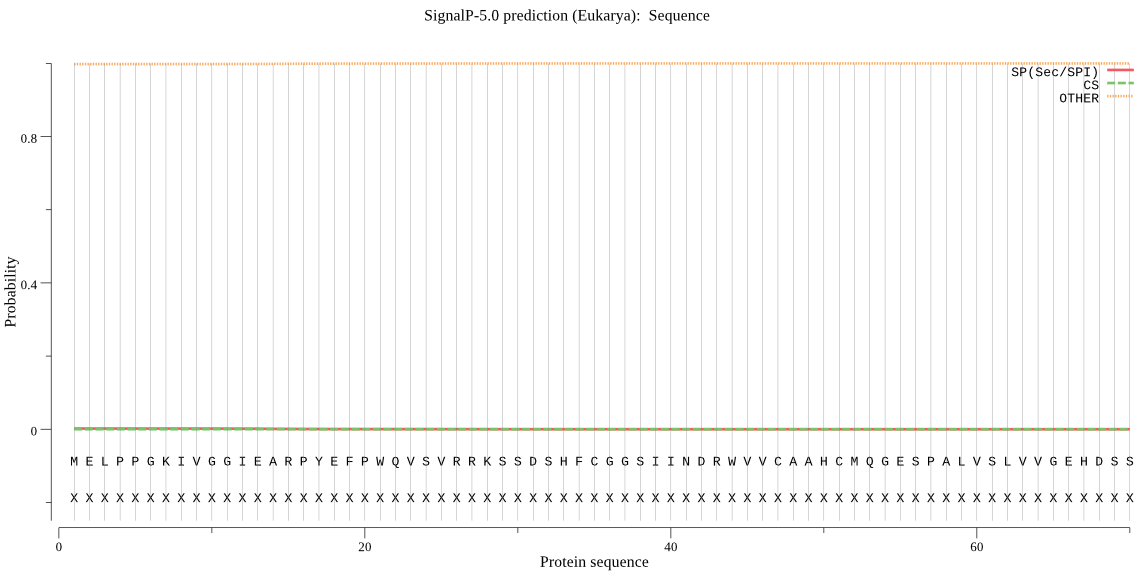

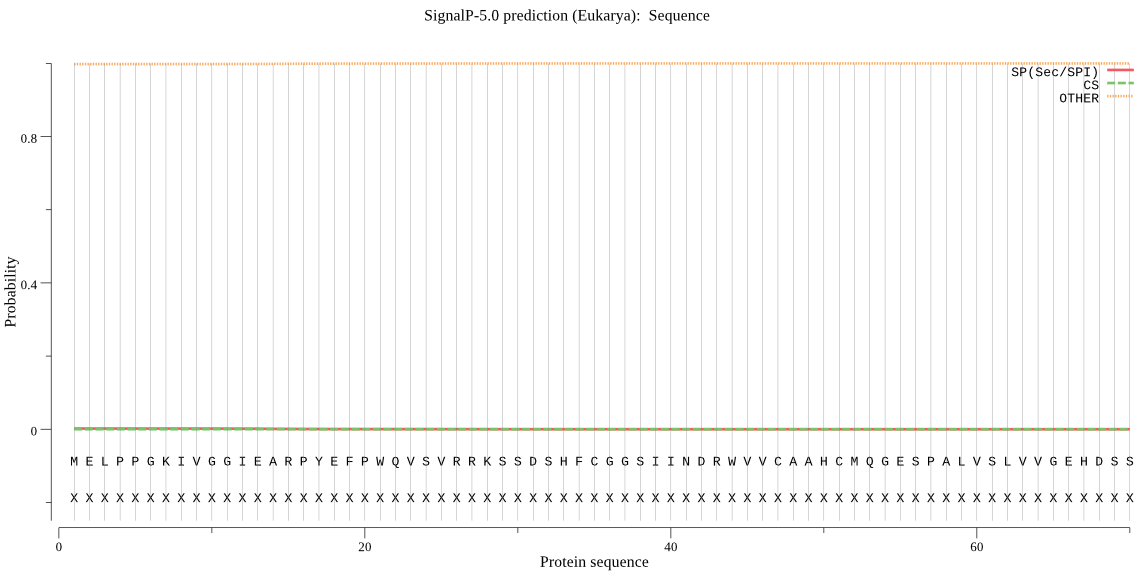

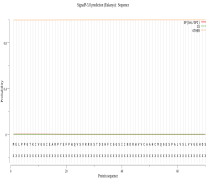


(25)

(26)

(27)

(28)

**
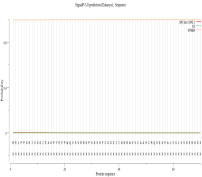

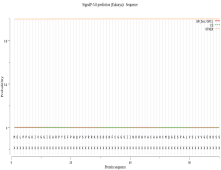

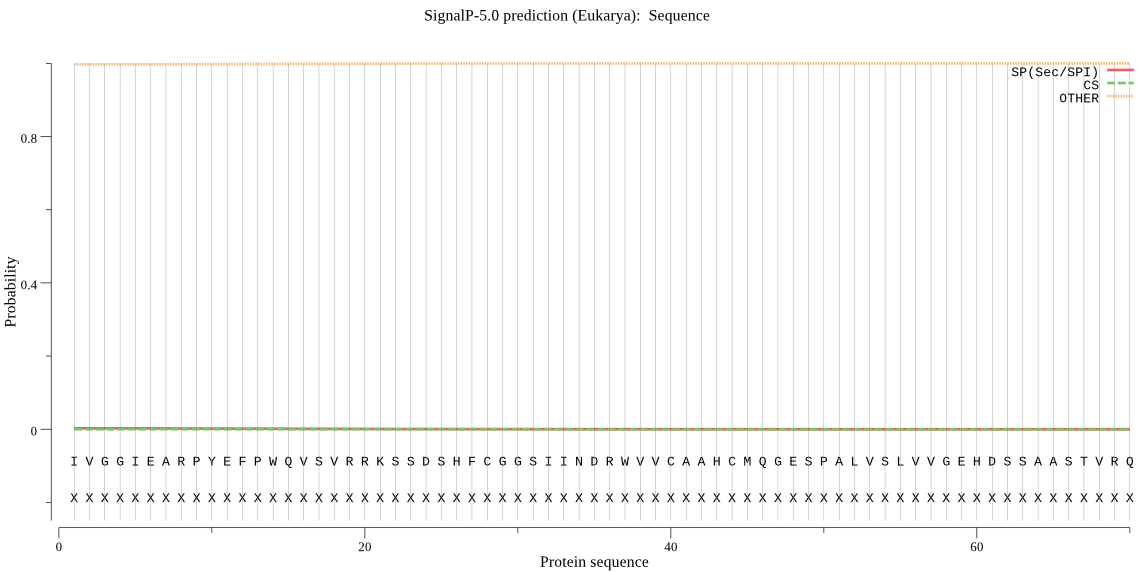
**

(29)

(30)

(31)

**Supplemental Figure 1**

(1)

(2)

(3)

(4)

(5)

(6)

(7)

(8)

(9)

(10)

(11)

(12)

(13)

(14)

(15)

(16)


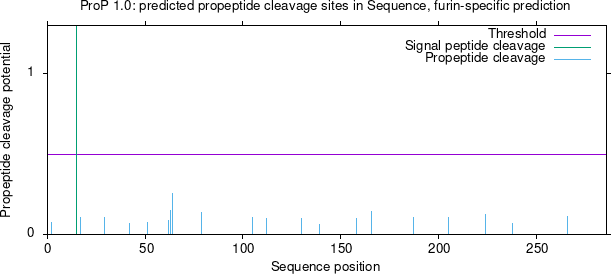

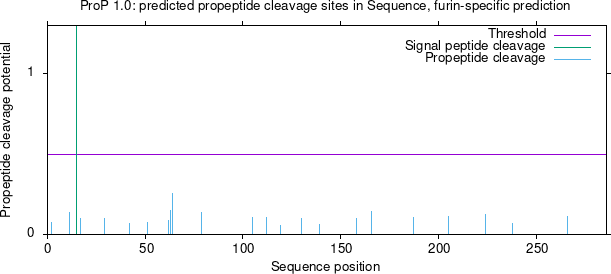

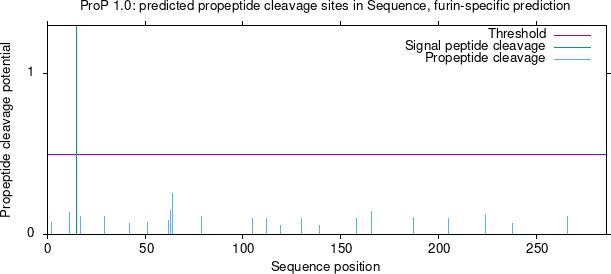

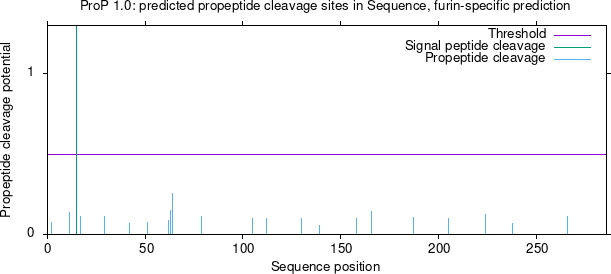

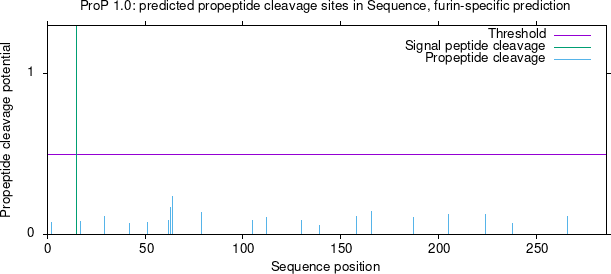

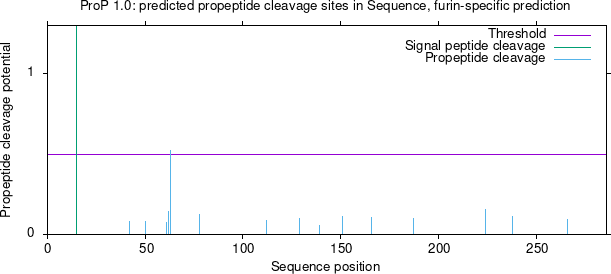

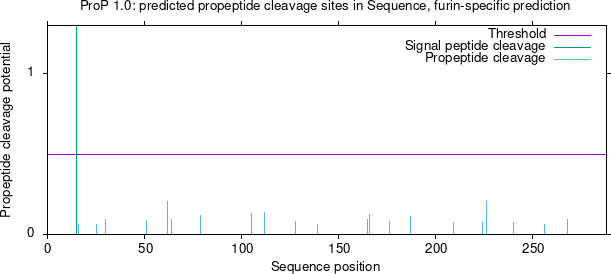

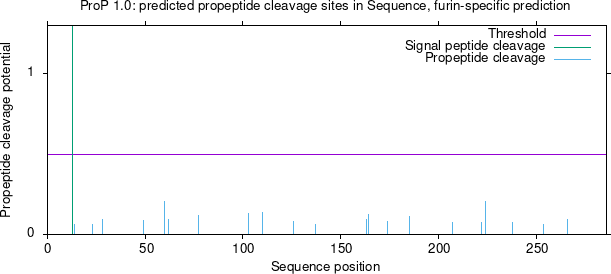

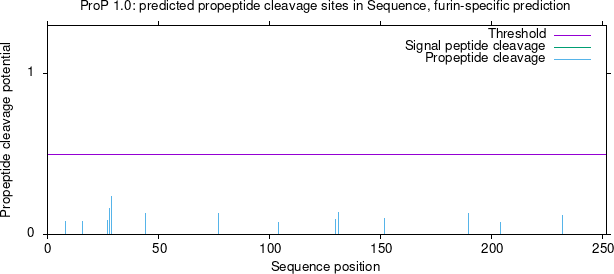

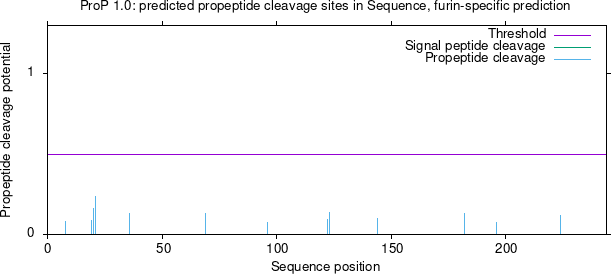

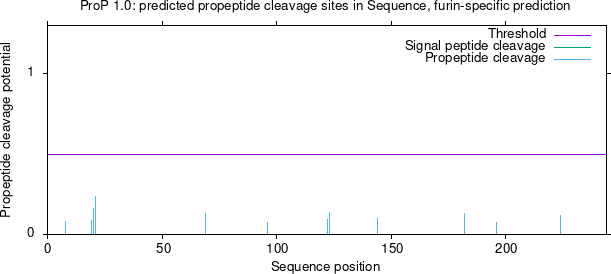

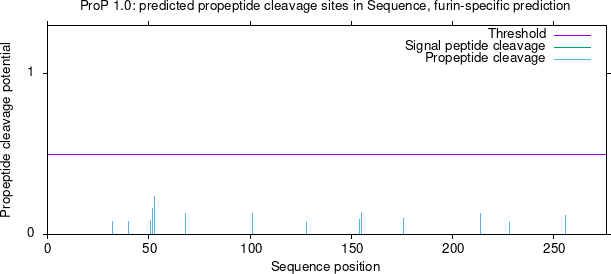

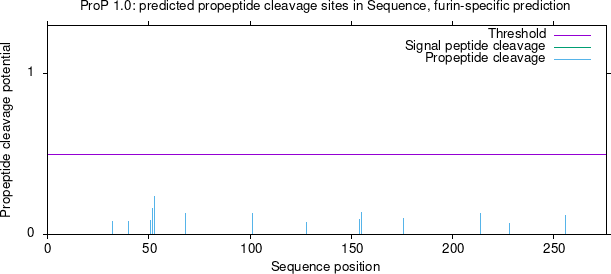

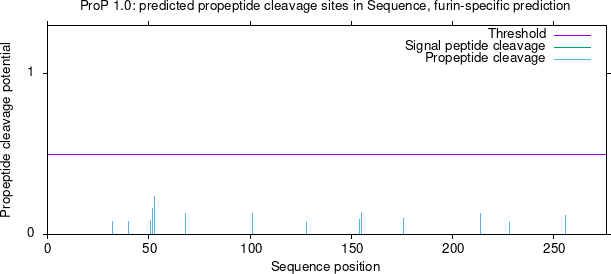

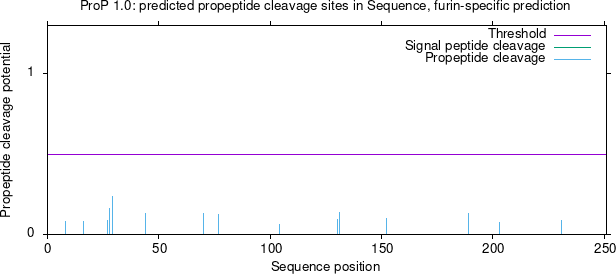

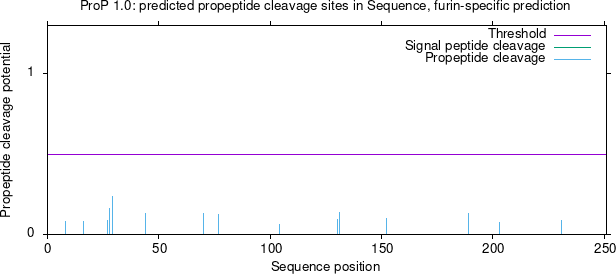


(17)

18)

(19)

(20)

(21)

(22)

(23)

(24)

(25)

(26)

(27)

(28)

(29)

(30)

(31)


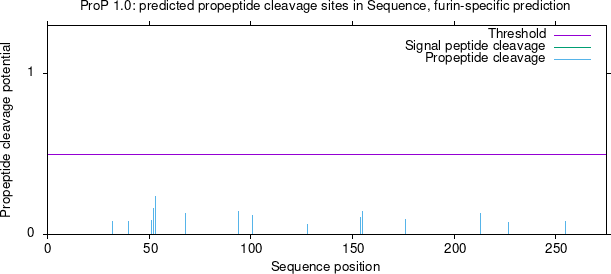

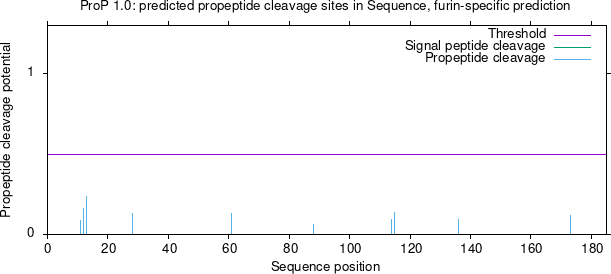

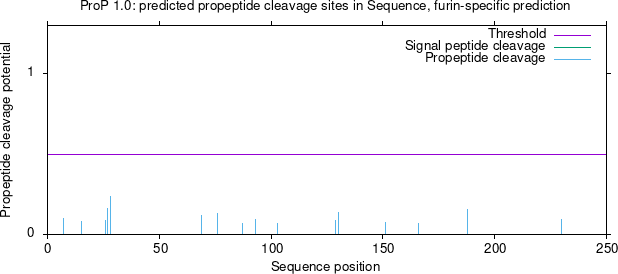

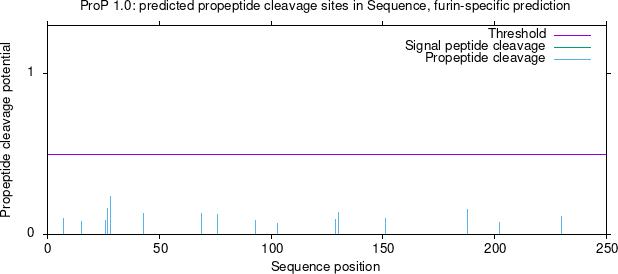

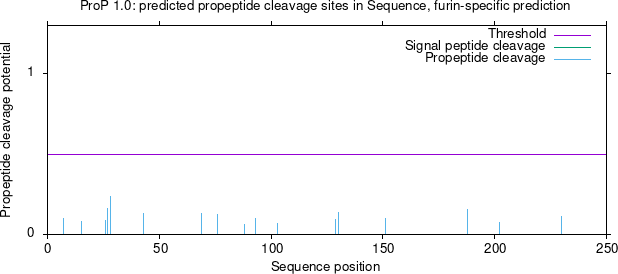

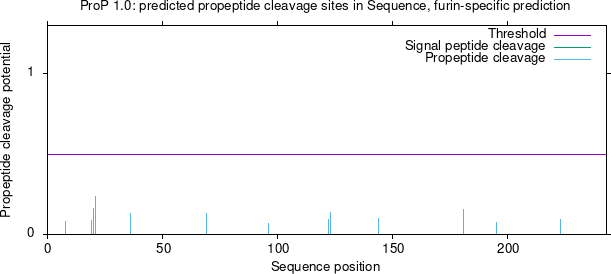

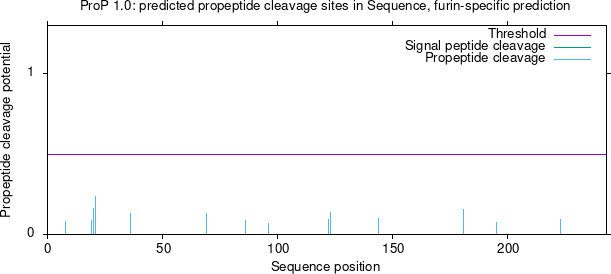

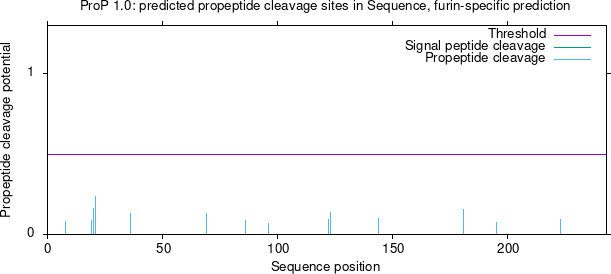

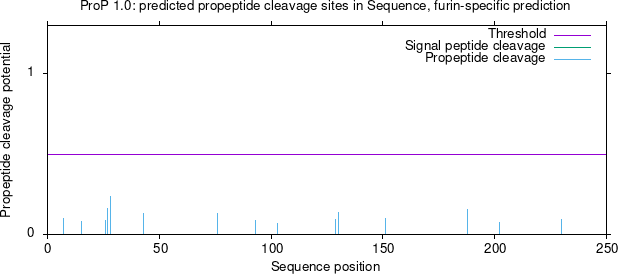

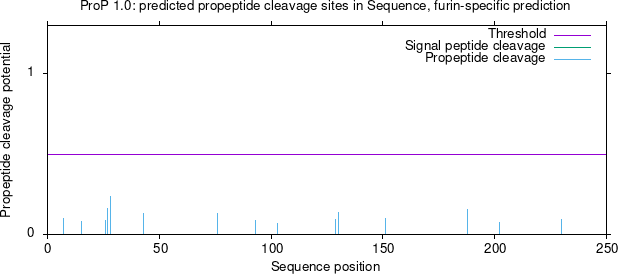

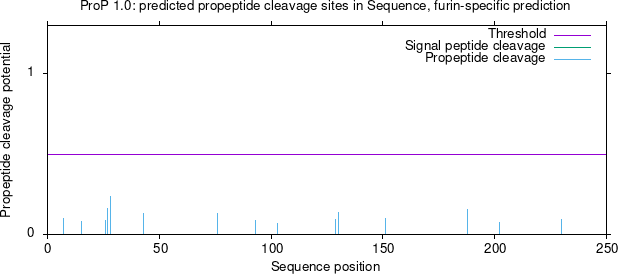

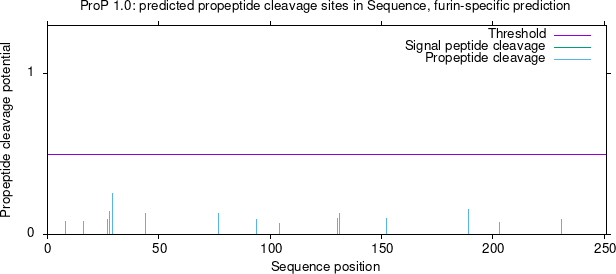

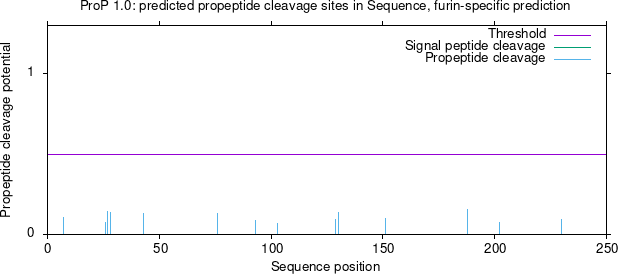

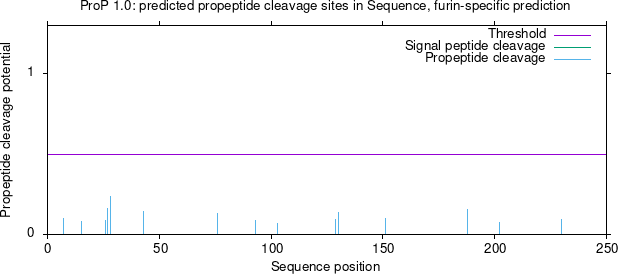

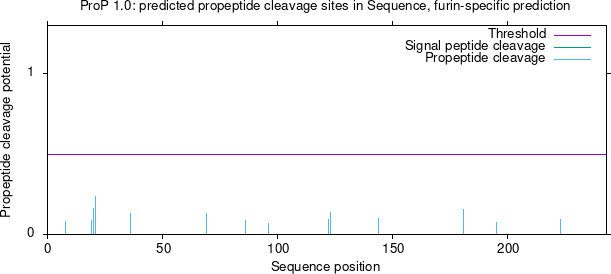


**Supplemental Figure 2**

(1)

(2)

(3)

(4)

(5)

(6)

(7)

(8)

(9)

(10)

(11)

(12)

(13)

(14)

(15)

(16)


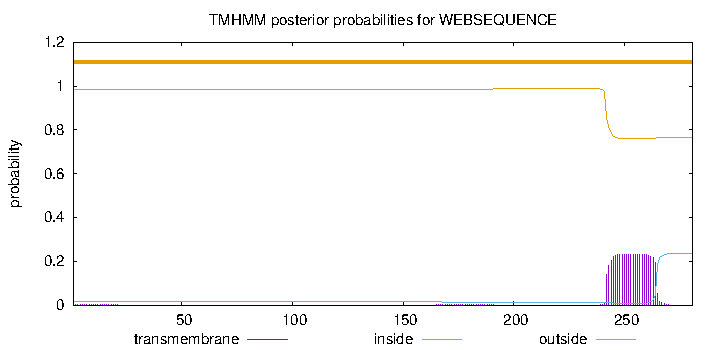

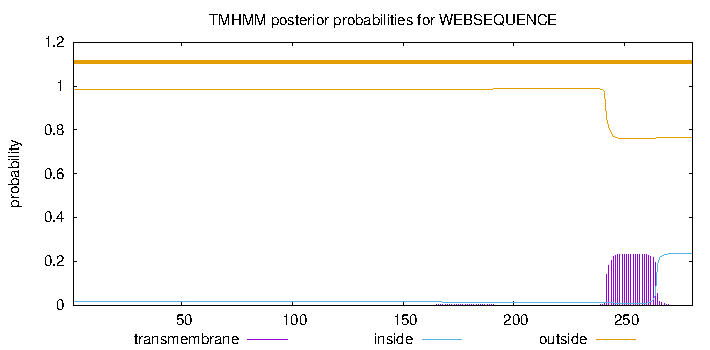

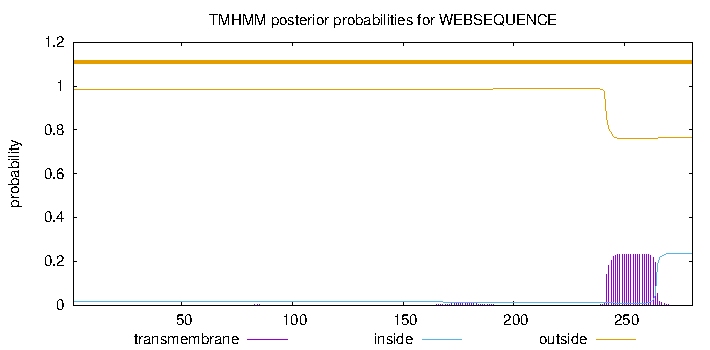

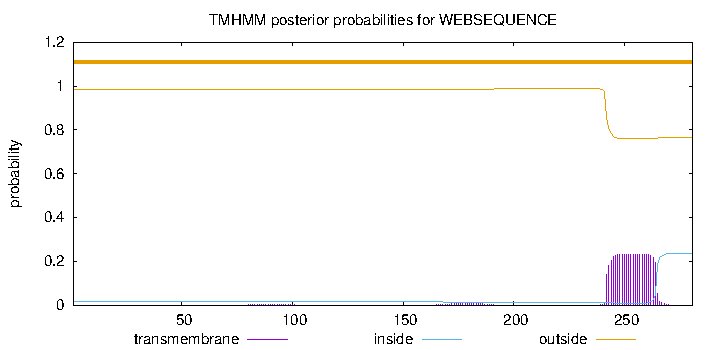

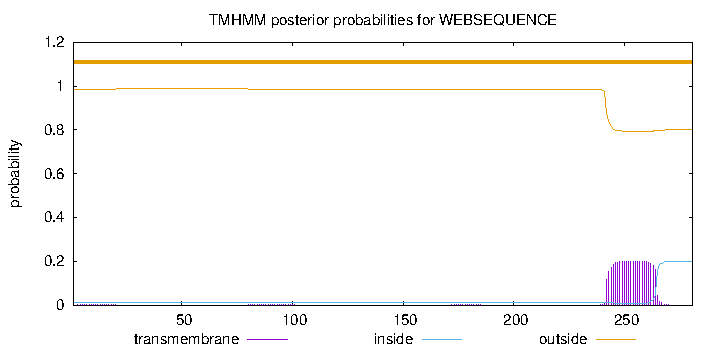

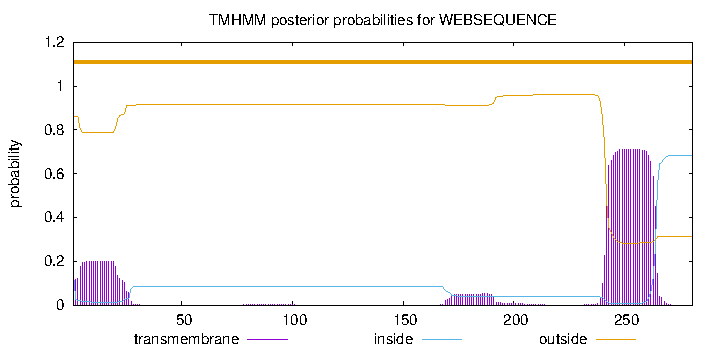

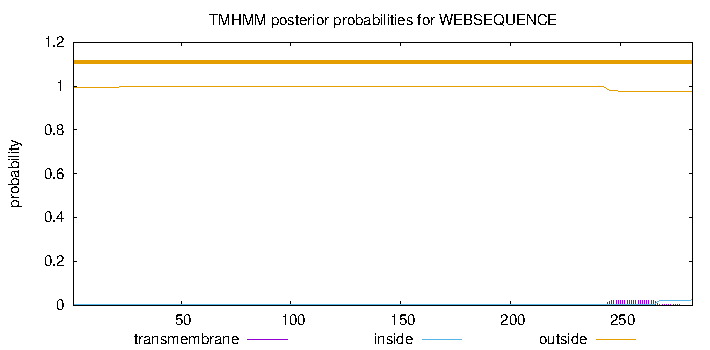

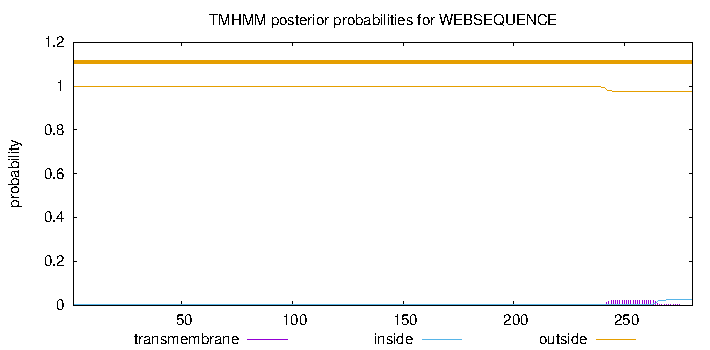

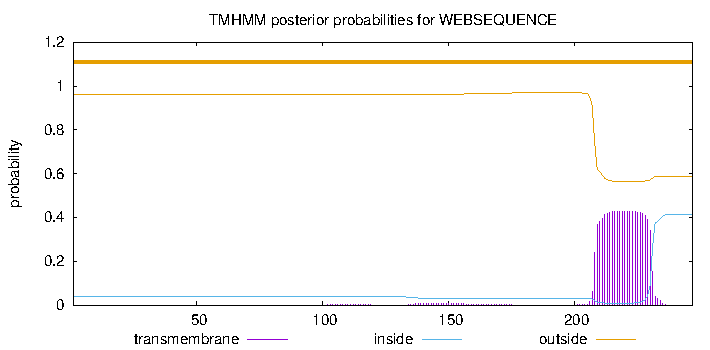

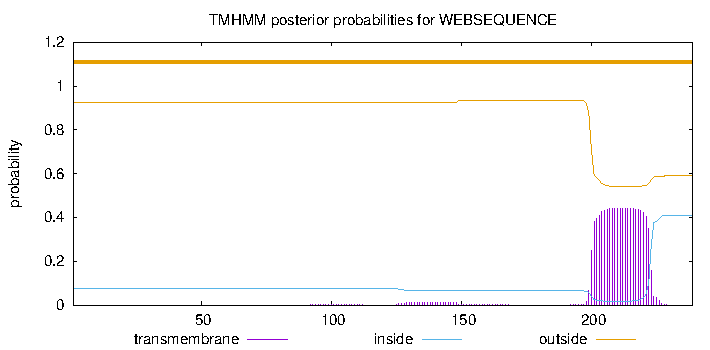

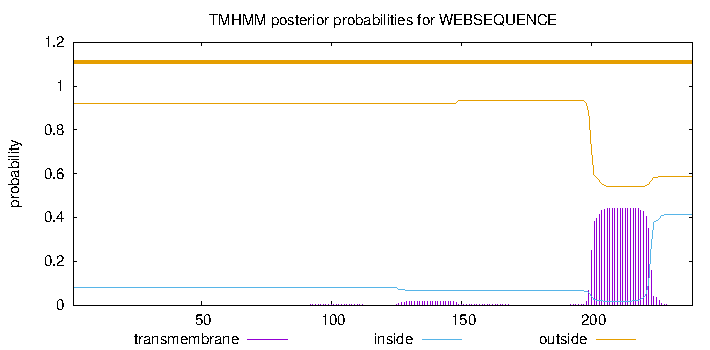

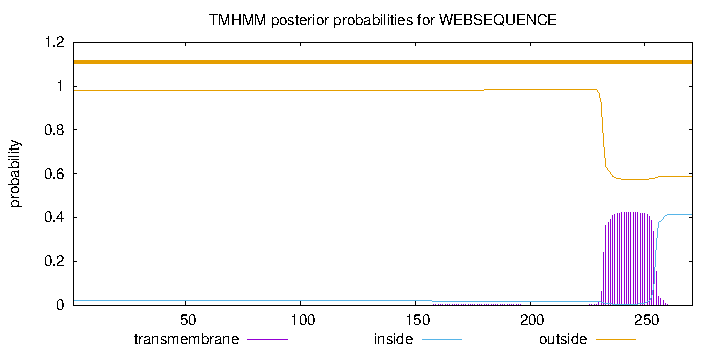

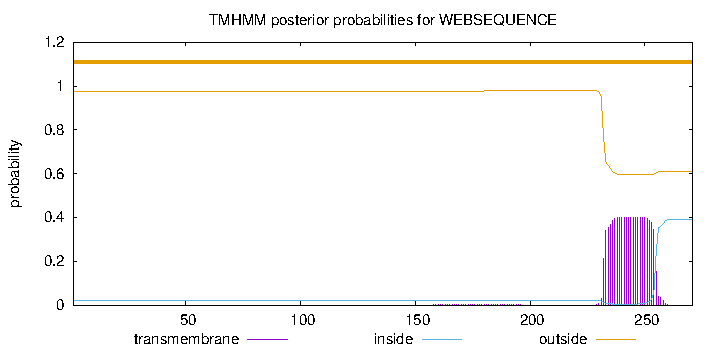

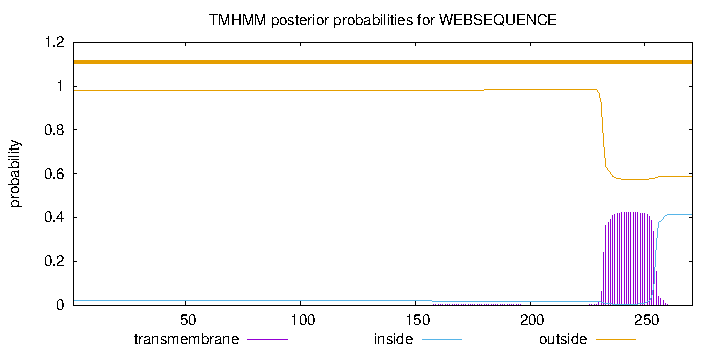

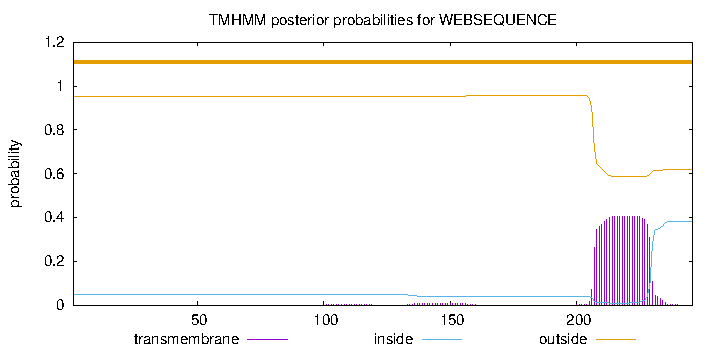

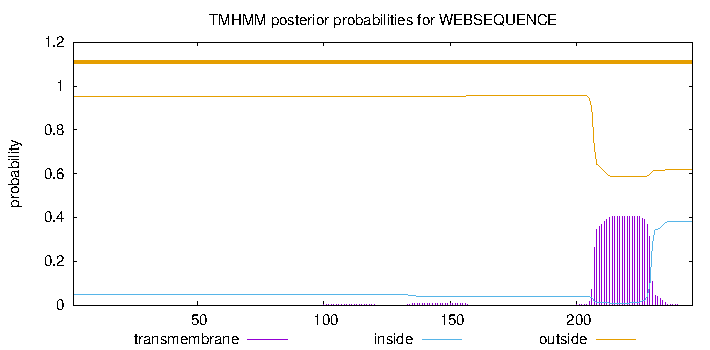


(17)

(18)

(19)

(20)

(21)

(22)

(23)

(24)

(25)

(26)

(27)

(28)

(29)

(30)

(31)


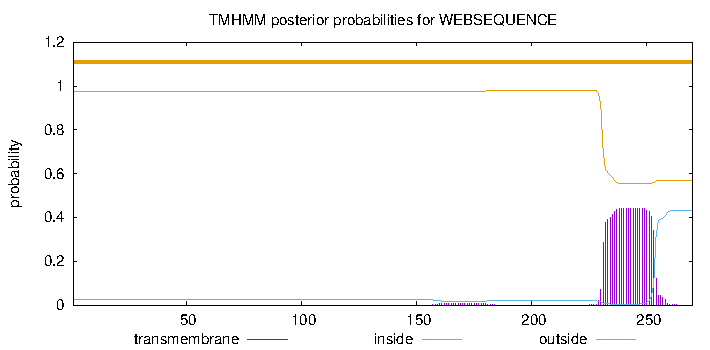

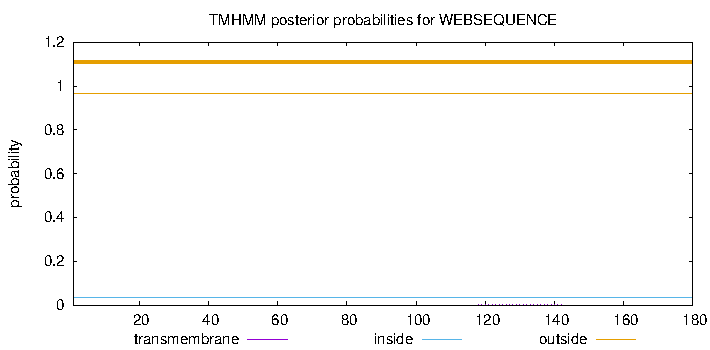

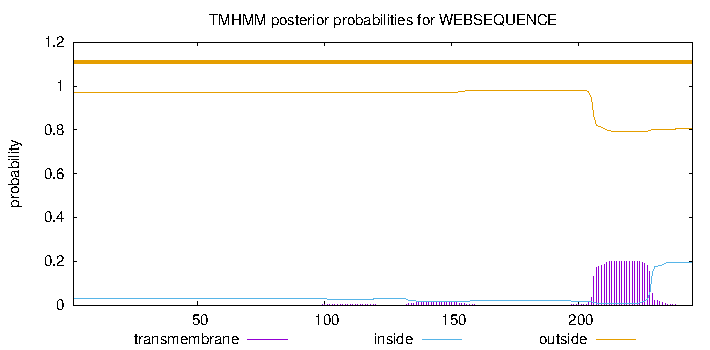

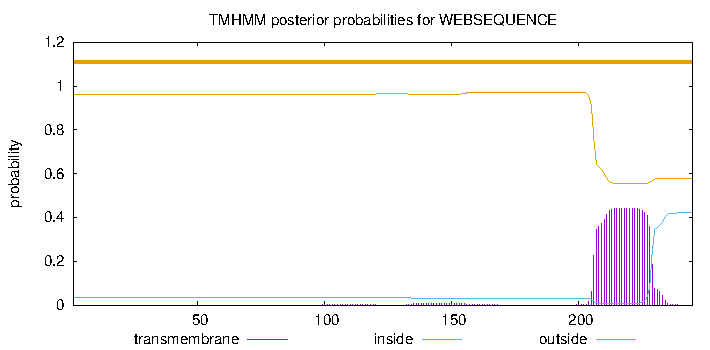

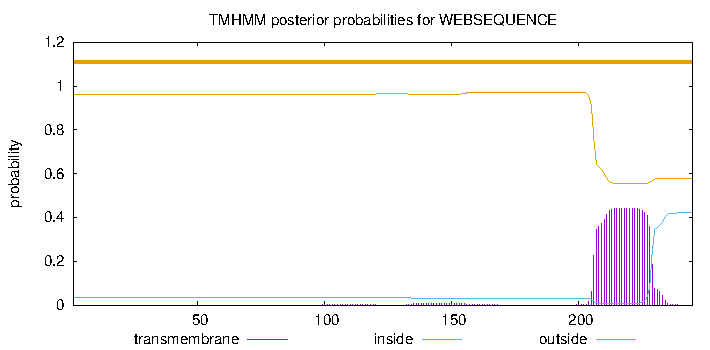

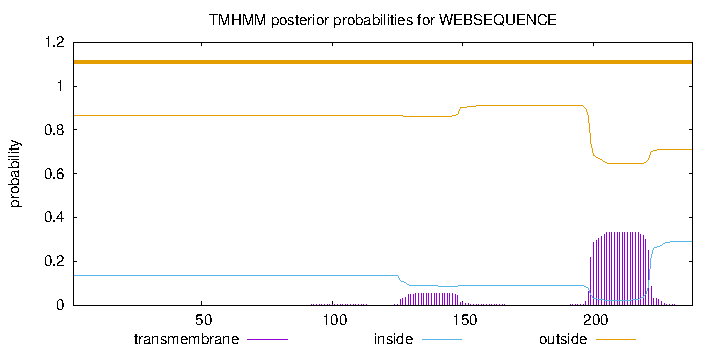

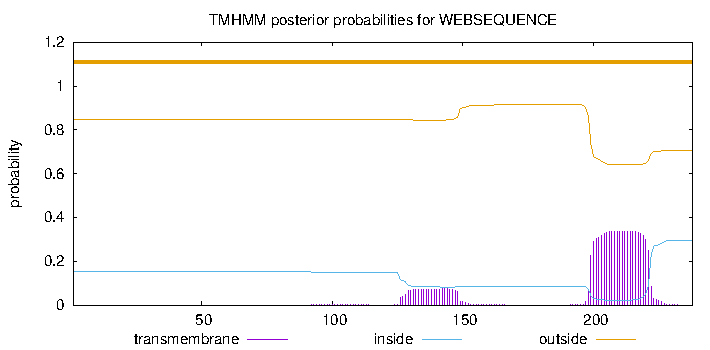

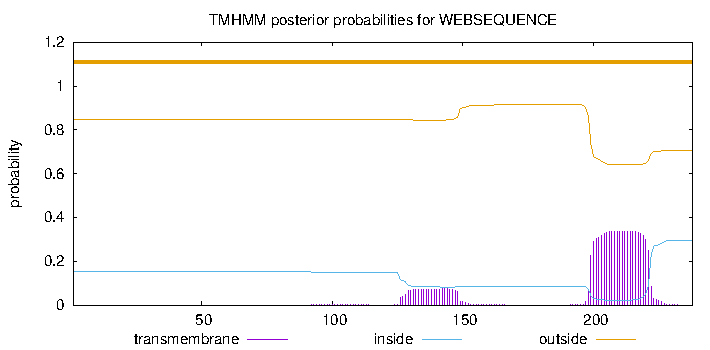

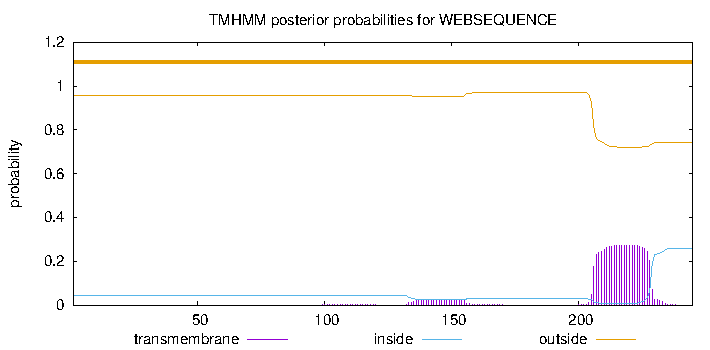

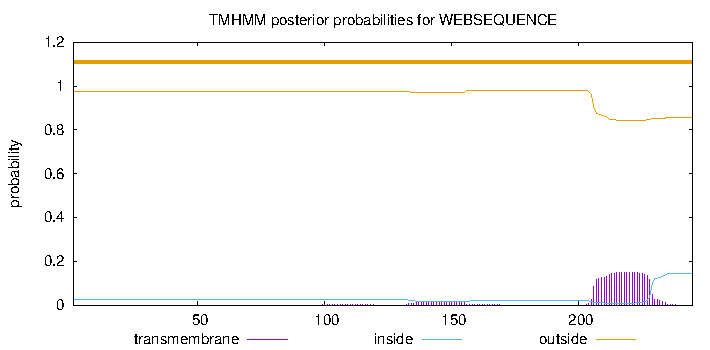

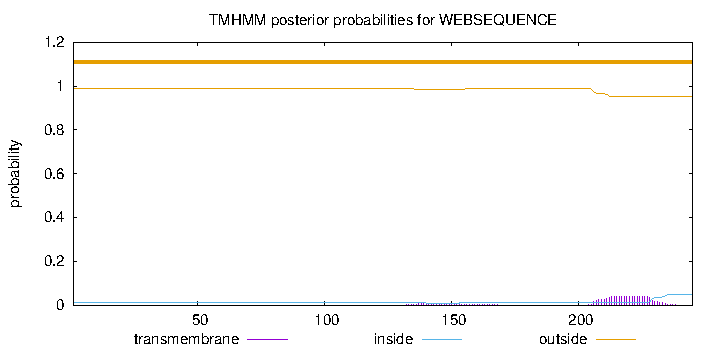

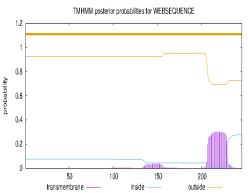

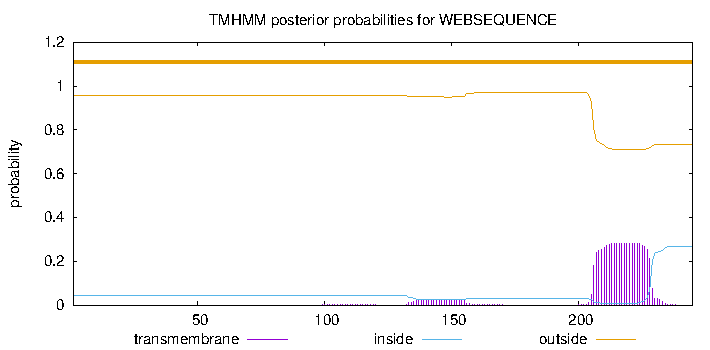

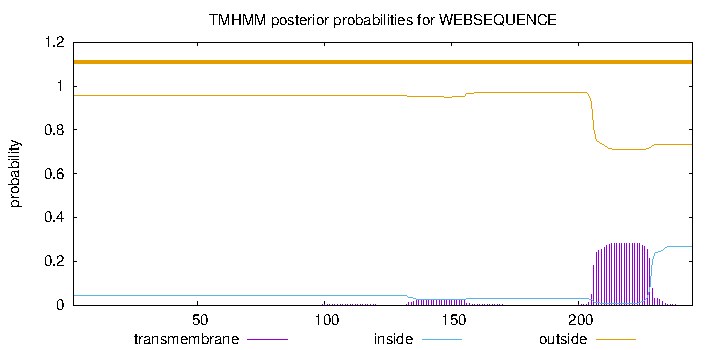

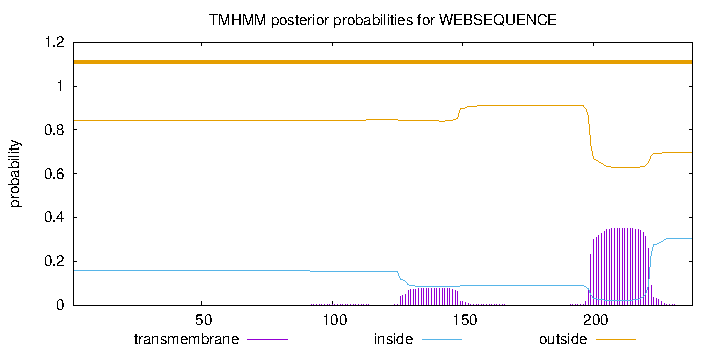


**Supplemental Figure 3**

(1)

(2)

(3)

(4)

(5)

(6)

(7)

(8)

(9)

(10)

(11)

(12)

(13)

(14)

(15)

(16)


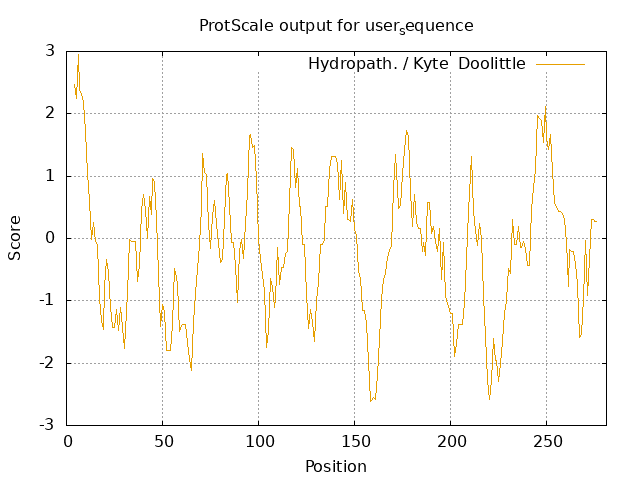

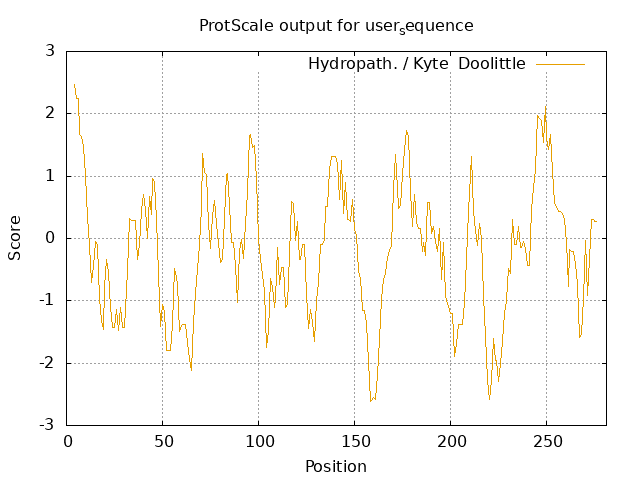

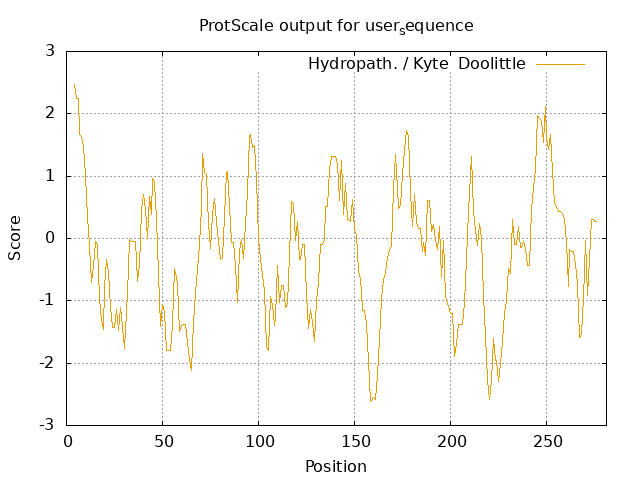

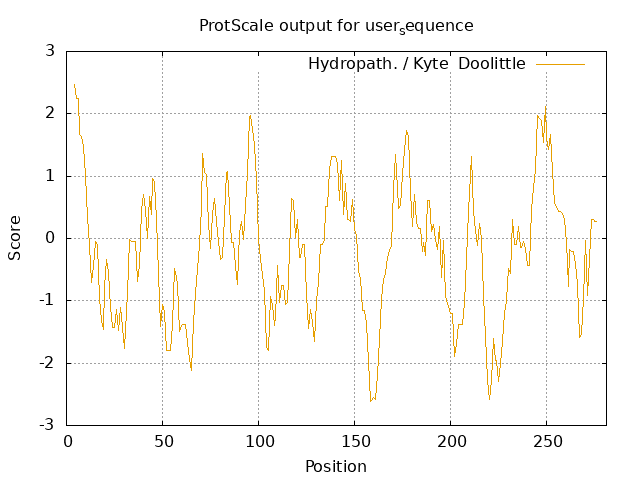

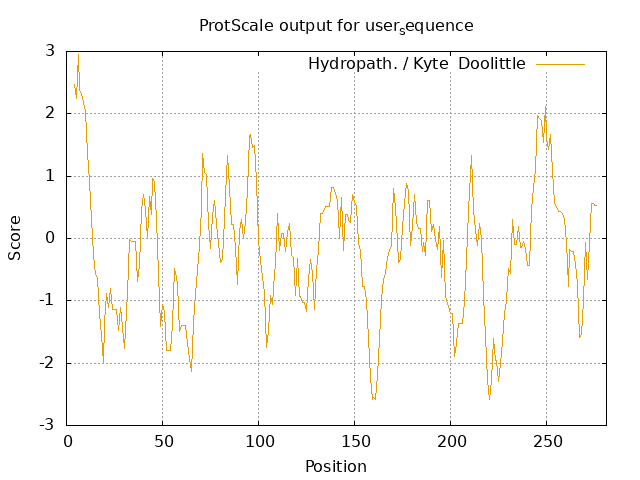

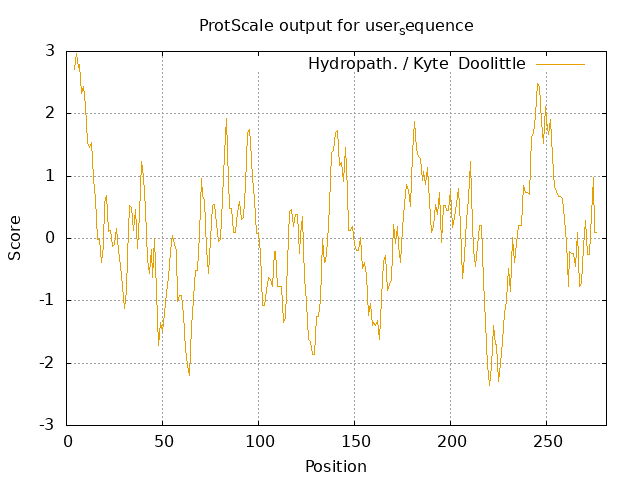

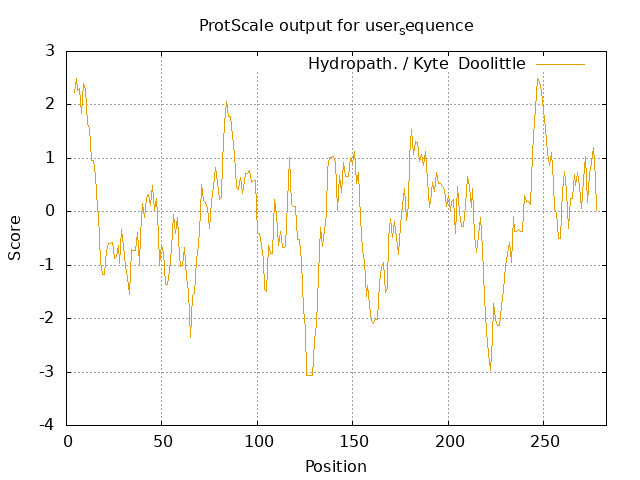


(17)

(18)

(19)

(20)

(21)

(22)

(23)

(24)

(25)

(26)

(27)

(28)

(29)

(30)

(31)

**Supplemental Figure 4**

(1)

(2)

(3)

(4)

(5)

(6)

(7)

(8)

(9)

(10)

(11)

(12)

(13)

(14)

(15)

(16)

(17)

(18)

(19)

(20)

(21)

(22)

(23)

(24)

(25)

(26)

(27)

(28)

(29)

(30)

(31)

**Supplemental Figure 5**

(1)

(2)

(3)

(4)

(5)

(6)

(7)

(8)

(9)

(10)

(11)

(12)

(13)

(14)

(15)

(16)

(17)

18)

(19)

(20)

(21)

(22)

(23)

(24)

(25)

(26)

(27)

(28)

(29)

(30)

(31)

**Supplemental Figure 6**

(1)

(2)

(3)

(4)

(5)

(6)

(7)

(8)

(9)

(10)

(11)

(12)

(13)

(14)

(15)

(16)

(17)

18)

(19)

(20)

(21)

(22)

(23)

(24)

(25)

(26)

(27)

(28)

(29)

(30)

(31)

**Supplemental Figure 7**

(1)

(2)

(3)

(4)

(5)

(6)

(7)

(8)

(9)

(10)

(11)

(12)

(13)

(14)

(15)

(16)

(17)

(18)

(19)

(20)

(21)

(22)

(23)

(24)

(25)

(26)

(27)

(28)

(29)

(30)

(31)

**Supplemental Figure 8**

| Supplemental table 1 The prediction of subcellular localization of the selected 31 lumbrokinase sequences | | | | | | | | | |
| --- | --- | --- | --- | --- | --- | --- | --- | --- | --- |
| Number | Genbank ID | nuclear | cytoplasmic | mitochondrial | endoplasmic reticulum | vacuolar | Golgi | vesicles of secretory system | extracellular(including cell wall) |
| 1 | BAL43192 |  |  |  | 11.1% | 11.1% |  |  | 77.8% |
| 2 | BAL43191 | 17.4% | 30.4% | 39.1% |  | 4.3% |  |  | 8.7% |
| 3 | BAL43189 | 17.4% | 30.4% | 30.4% |  | 4.3% |  |  | 17.4% |
| 4 | BAL43190 | 17.4% | 30.4% | 30.4% |  | 4.3% |  |  | 17.4% |
| 5 | BAL43193 |  |  |  | 11.1% | 11.1% | 11.1% |  | 66.7% |
| 6 | CAA11132 |  |  | 11.1% | 11.1% | 11.1% |  |  | 66.7% |
| 7 | T2204P LUKA |  | 11.1% | 11.1% |  | 22.2% |  |  | 55.6% |
| 8 | T2205P LUKA |  | 11.1% | 11.1% |  | 22.2% |  |  | 55.6% |
| 9 | AAR13224 | 26.1% | 56.5% | 17.4% |  |  |  |  |  |
| 10 | ABQ23217 | 13.0% | 65.2% | 17.4% |  |  |  | 4.3% |  |
| 11 | AAQ13828 | 13.0% | 65.2% | 17.4% |  |  |  | 4.3% |  |
| 12 | AAA96502 | 8.7% | 69.6% | 13.0% | 4.3% |  |  | 4.3% |  |
| 13 | AAN28692 | 8.7% | 69.6% | 13.0% | 4.3% |  |  | 4.3% |  |
| 14 | AAL28118 | 13.0% | 69.6% | 13.0% |  |  |  | 4.3% |  |
| 15 | BAB40768 | 26.1% | 56.5% | 17.4% |  |  |  |  |  |
| 16 | AAN78282 | 26.1% | 56.5% | 17.4% |  |  |  |  |  |
| 17 | AAA96503 | 13.0% | 69.6% | 13.0% |  |  |  | 4.3% |  |
| 18 | AAL27616 | 17.4% | 56.5% | 21.7% |  | 4.3% |  |  |  |
| 19 | AXK90150 | 21.7% | 65.2% | 13.0% |  |  |  |  |  |
| 20 | ATP16189 | 26.1% | 56.5% | 17.4% |  |  |  |  |  |
| 21 | ARD24433 | 26.1% | 56.5% | 17.4% |  |  |  |  |  |
| 22 | 1YM0_A | 17.4% | 60.9% | 17.4% |  |  |  | 4.3% |  |
| 23 | AIC77168 | 17.4% | 60.9% | 17.4% |  |  |  | 4.3% |  |
| 24 | ABB19359 | 17.4% | 60.9% | 17.4% |  |  |  | 4.3% |  |
| 25 | AHY19039 | 17.4% | 69.6% | 13.0% |  |  |  |  |  |
| 26 | BAB40767 | 17.4% | 69.6% | 8.7% |  | 4.3% |  |  |  |
| 27 | QBA57435 | 17.4% | 69.6% | 8.7% |  | 4.3% |  |  |  |
| 28 | AAT74900 | 26.1% | 56.5% | 17.4% |  |  |  |  |  |
| 29 | AAT74899 | 13.0% | 69.6% | 13.0% |  |  |  | 4.3% |  |
| 30 | ABA43718 | 21.7% | 69.6% | 8.7% |  |  |  |  |  |
| 31 | ABW04903 | 17.4% | 60.9% | 17.4% |  |  |  | 4.3% |  |

| Supplemental table 2. NetCTL-1.2 predictions of LUKA using MHC supertype A1 | | | |
| --- | --- | --- | --- |
| Position | Sequence | Score | significants level |
| 118 | DSIFVNEDY | 1.397 | 0.01 |
| 154 | CAPDPDNDY | 0.9522 | 0.05 |
| 180 | ICCPAVLRY | 0.8454 | 0.05 |
| 199 | CADLYNPTV | 0.8454 | 0.05 |
| 217 | TTDNTGQKH | 1.597 | 0.01 |
| Note: score>1.25, specificity=0.993; score>0.75, specificity=0.970. | | | |
